# The Small Molecule H89 Facilitates Mesenchymal Stem Cell‐derived Extracellular Vesicle Release and Optimizes Therapeutic Efficacy in Liver Regeneration

**DOI:** 10.1002/jev2.70285

**Published:** 2026-05-09

**Authors:** Yu Fu, Yi Ma, Jiajun Zhang, Liwei Liang, Ting Li, Zeyi Guo, Zhongzhe Li, Lei Feng, Yi Wang, Guolin He, Shao Li, Yang Li, Xiaoping Xu, Hui Liao, Yi Gao

**Affiliations:** ^1^ Department of Hepatobiliary Surgery II GuangDong Engineering Technology Research Center of Artificial Organ and Tissue Engineering Guangzhou Clinical Research and Transformation Center for Artificial Liver Institute of Regenerative Medicine General Surgery Center Zhujiang Hospital Southern Medical University Guangzhou Guangdong China; ^2^ State Key Laboratory of Organ Failure Research Southern Medical University Guangzhou China; ^3^ Guangdong Provincial Key Laboratory for Prevention and Control of Major Liver Diseases Southern Medical University Guangzhou China; ^4^ Department of Hepatobiliary Surgery the Fifth Affiliated Hospital Southern Medical University Guangzhou Guangdong China; ^5^ Department of Hepatobiliary Surgery the Third Affiliated Hospital of Guangzhou Medical University Guangzhou Guangdong China; ^6^ Department of Hepatic Surgery IV Eastern Hepatobiliary Surgery Hospital Naval Medical University (Second Military Medical University) Shanghai China; ^7^ Department of Hepatic‐Biliary‐Pancreatic Surgery the Affiliated Hospital of Guizhou Medical University Guiyang Guizhou China; ^8^ Shanxi Province Cancer Hospital/ ShanxiHospital Affiliated to Cancer Hospital Chinese Academy of MedicalSciences/Cancer Hospital Affiliated to Shanxi Medical University Taiyuan Shanxi China

**Keywords:** amphisomes, extracellular vesicles, GABARAPL1, H89, hepatic stellate cells, liver regeneration, mesenchymal stem cells, miR‐29a, RELA

## Abstract

The role of human umbilical cord mesenchymal stem cell‐derived extracellular vesicles (hUCMSC‐EVs) in liver regeneration is promising, yet their clinical translation is hampered by insufficient production. Current strategies targeting their secretion are inefficient and lack a clear mechanistic understanding. We isolated and characterized hUCMSC‐EVs pretreated with the H89 and other mTORC1 inhibitors. Our findings revealed that H89 effectively enhances the secretion of hUCMSC‐EVs across diverse cell types, demonstrating universal efficacy. Importantly, H89 upregulates GABARAPL1 expression, a key negative regulator of the PKA/mTORC1 pathway, to inhibit mTORC1 activity and promote the formation of amphisomes and SNARE‐mediated hUCMSC‐EVs release. Furthermore, EVs derived from H89‐pretreated hUCMSCs (H‐EVs) exhibited altered cargo composition, significantly increased proliferative activity, and potentiated liver regeneration via the RELA/miR‐29a axis, which regulates the homeostasis of hepatic stellate cells. Our results highlight that H89 enhances hUCMSC‐EV secretion through mTORC1 inhibition, with the resulting benefits for liver regeneration mediated by the RELA/miR‐29a network. These findings demonstrate the great promise of H89 in EV‐based liver regeneration, offering a promising platform for clinical translation.

## 1 Introduction

Human umbilical cord mesenchymal stem cell‐derived extracellular vesicles (hUCMSC‐EVs) exhibit significant therapeutic potential in promoting liver regeneration because of their unique endogenous regulatory properties (Xu et al. 2016). Previous studies have demonstrated that hUCMSCs can effectively suppress the inflammatory cytokine storm and show remarkable efficacy in treating a rat model of 90% hepatectomy‐induced acute liver failure (ALF) (Yi et al. 2024). Further studies have shown that EVs can promote liver regeneration and significantly improve liver function in acute liver injury models (Li et al. 2022). Additionally, hUCMSC‐EVs are promising for clinical applications, including in immune modulation and serving as drug delivery vehicles. As drug carriers, they not only enhance therapeutic outcomes but also significantly reduce treatment‐related side effects (Cully 2021; Elsharkasy et al. 2020).

hUCMSC‐EVs have emerged as important tools in regenerative medicine, immunotherapy, and targeted therapy. However, their translation into clinical applications remains slow, primarily because the yield of EVs is insufficient to meet clinical demands. Current pretreatment strategies for stimulating the release of MSC‐derived EVs can be divided into four main categories: optimization of cell culture conditions; genetic modification of MSCs; physical stimulation; and compound stimulation. The optimization of cell culture conditions primarily involves increasing the cell culture surface area or scaling up production, including the use of hollow‐fiber reactor cell expansion technology (Feng et al. 2023). Genetic modification of MSCs involves the overexpression of specific genes, such as HIF‐1α (Sun et al. 2020) and Shp2 (Zhang et al. 2021). Physical stimulation methods include LED light irradiation (Ruan et al. 2022), low‐intensity ultrasound pulses (Zheng et al. 2023b), and hypoxic culture conditions (Gupta et al. 2022) during cell culture. However, these physical stimulation methods often require specialized equipment, may induce widespread cellular responses, lack specificity for targeted pathways, and are associated with high pretreatment costs and unstable outcomes. In contrast, genetic modification of MSCs while potentially more effective in some cases, also faces challenges similar to those associated with physical stimulation methods, such as operational complexity and ethical concerns in clinical applications, and requires expertise in gene editing technologies. For compound stimulation, commonly used methods include the application of inflammatory cytokines such as INF‐γ (Serejo et al. 2019), TNF‐α (Wang et al. 2017), and LPS (Li et al. 2021). These approaches are convenient and safe, allowing for flexible and reversible modulation of cellular biological effects through dose adjustments. However, compound intervention strategies face two critical challenges. First, large macromolecular biological factors regulate cell behavior through complex multitarget networks, making it difficult to precisely modulate EV secretion pathways. Second, while small molecules with molecular weights less than 900 Da (e.g., metabolic regulators) exhibit excellent cell membrane permeability and can directly act on intracellular targets to achieve specific modulation, their incremental efficiency in enhancing EV secretion (typically less than 2‐fold) remains insufficient for clinical translation.

Extracellular vesicles (EVs) are generated from multivesicular bodies (MVBs) which originate from intraluminal vesicles (ILVs). Autophagy is the major intracellular degradation system through which cytoplasmic materials are delivered to and degraded in the lysosomes (Kalluri and LeBleu 2020). There are significant correlations between the secretion of EVs and the regulation of autophagy (Zou et al. 2019). H89 is a widely used PKA inhibitor. In addition, H89 is closely related to autophagy regulation (Byun et al. 2020). Pretreating MSCs with H89 may affect EV secretion. The exact mechanism underlying the therapeutic effect of MSC‐derived EVs (MSC‐EVs) remains largely obscure and is a topic of intensive investigation.

The present study was designed to investigate the effects of H89 on MSC‐EVs and the mechanisms of EV secretion. To test whether H89 induces EV production, we compared EV secretion from normal and H89‐pretreated MSCs. To further confirm the therapeutic effect of EVs derived from H89‐pretreated hUCMSCs (H‐EVs) and EVs derived from normal hUCMSCs (C‐EVs), we performed in vitro and in vivo experiments. Moreover, proteomics analysis revealed that H89 affects the protein cargoes in H‐EVs, accounting for their improved therapeutic effects on liver regeneration. In summary, our study suggests that H89 plays an important role in facilitating the secretion of EVs and optimizing the therapeutic potential of EVs in liver regeneration.

## 2 Results

### 2.1 H89 Promotes MSC‐EV Secretion Quantitatively

**FIGURE 1:**
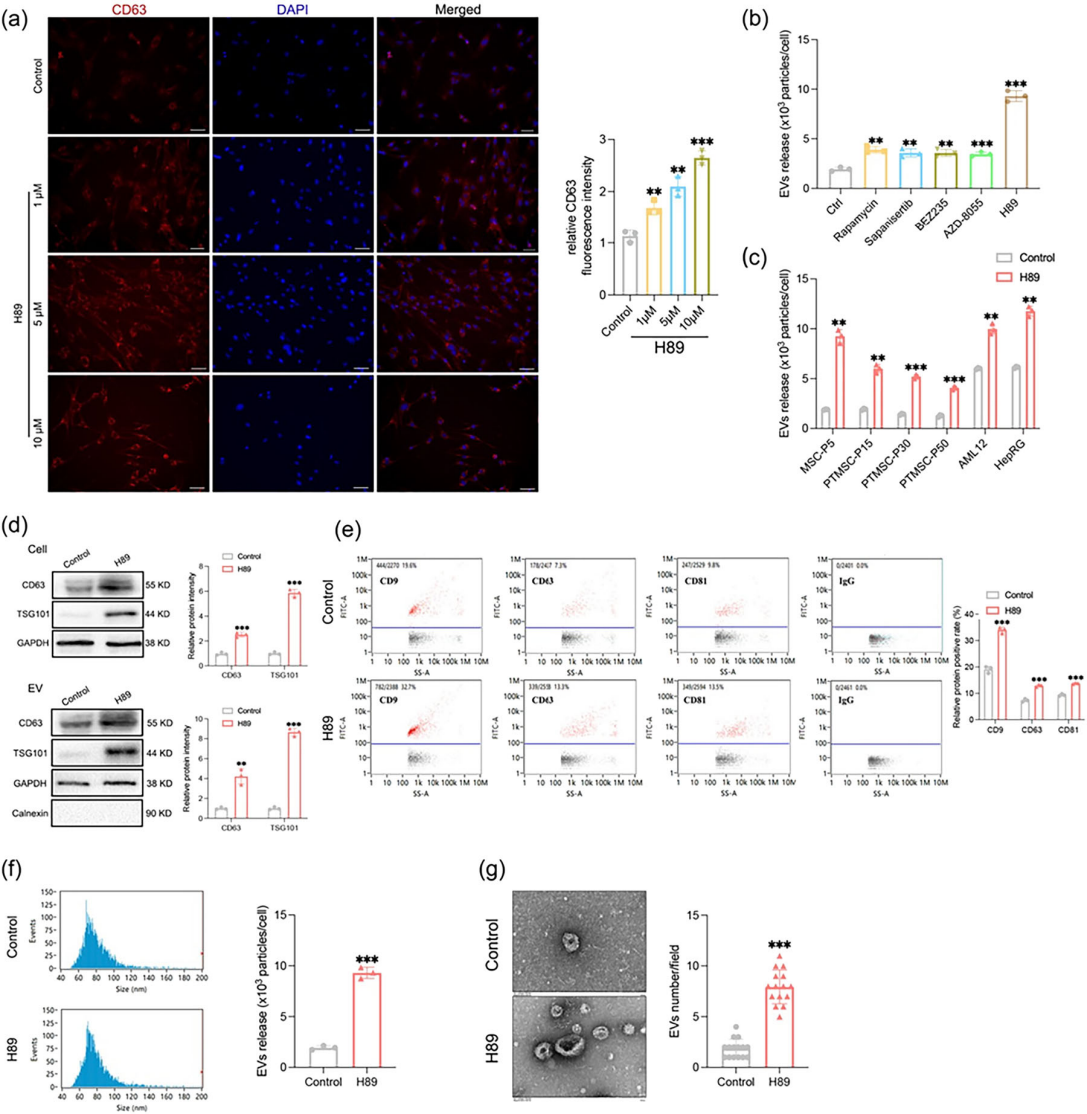
H89 promotes extracellular vesicle (EV) production and release in hUCMSCs. (a) hUCMSCs were treated with 1 µM, 5 µM, or 10 µM H89 for 24 h, while cells in the control group received an equal volume of phosphate‐buffered saline (PBS). Immunofluorescence staining was used to visualize CD63 (red), and DAPI was used to stain nuclei (blue). The fluorescence intensity was quantified using ImageJ software (*n* = 3). Scale bar: 100 µm. (b) Quantitative analysis of extracellular vesicle (EV) concentration by nanoflow cytometry (NanoFCM) in hUCMSCs pretreated with 10 µM H89 or mTORC1 inhibitors, as shown in Figure S1. The exact fold‐change for the H89 treatment group was 4.87‐fold compared to the control. (c) NanoFCM quantification of EV secretion by AML12 cells, HepRG cells, MSCs, and PTMSCs after 48 h of treatment with 10 µM H89. (d) Western blot analysis of CD63 and TSG101 expression in cellular lysates and EV fractions isolated from hUCMSCs treated with PBS (negative control) or 10 µM H89 for 48 h. (e, f) NanoFCM quantification of the expression of CD63, CD9, and CD81 in EVs, along with the particle size distribution and concentration. (g) EVs were visualized by transmission electron microscopy (TEM). EV density was quantified in 15 random fields (*n* = 3). All the data are presented as the mean ± SD. Unpaired two‐tailed Student's t tests and One‐way ANOVA followed by Dunnett's multiple comparisons test were used to test for statistical significance. **p < 0.01, ***p < 0.001.

During the investigation of the effects of H89 on human umbilical cord mesenchymal stem cells (hUCMSCs), we observed a dose‐dependent increase in CD63 immunostaining intensity in treated group compared with the control group Figure 1a. Consistent with the immunofluorescence imaging, Western Blot analysis of cell and EV lysates confirmed a significant upregulation of CD63 and syntenin protein expression following H89 treatment Figure S1a, b. Given the predominant localization of CD63 to MVBs, which are the primary precursors of EVs, these findings suggest that H89 may modulate EV secretion pathways in hUCMSCs. Recent studies have shown that mTORC1 can hinder the release of EVs (Zou et al. 2019). H89 (chemical formula: C_20_H_20_BrN_3_O_2_S), as a commonly used PKA inhibitor, has been shown to regulate mTORC1 activity (Bai et al. 2024). Therefore, we hypothesized that H89 may promote the release of EVs by influencing autophagy. To determine whether mTORC1 inhibitors can promote EV release and to identify the most effective small molecules, we conducted a comparative analysis of the effects of various mTORC1 inhibitors on hUCMSC‐EV production. We designed experiments with different concentration gradients and pretreatment durations Figure S1c, d to determine the optimal dose and treatment time. All tested mTORC1 inhibitors promoted EV secretion, with the effect of the small molecule H89 being the most significant, achieving a 4.87‐fold increase compared with that in the control group Figure 1b. To further assess the generalizability of the EV‐promoting effect of H89, we pretreated various cell types with H89. The results showed that H89 significantly promoted EV release across multiple cell lines, indicating that H89 pretreatment has broad applicability for enhancing EV secretion Figure 1c.

Next, we pretreated hUCMSCs with H89 and detected the protein levels of the EV markers CD63 and TSG101 in cells and EV components Figure 1d. The protein levels of both CD63 and TSG101 were high in cell and EV lysates. Further NanoFCM analysis and TEM imaging also revealed that the production of hUCMSC EVs after H89 pretreatment was greater than that in the control group; additionally, H89 pretreatment did not affect the size or morphology of EVs, but did increase the concentration of EVs by 4.87‐fold Figure 1e–g, S1e–g. As H89 is a low‐cost, readily available, small‐molecule drug with a clear chemical formula and can promote cell proliferation at effective concentrations, we investigated whether H89 can be used to increase the production of hUCMSC EVs.

### 2.2 H89‐Induced Amphisomes Are Associated with EV Production

**FIGURE 2:**
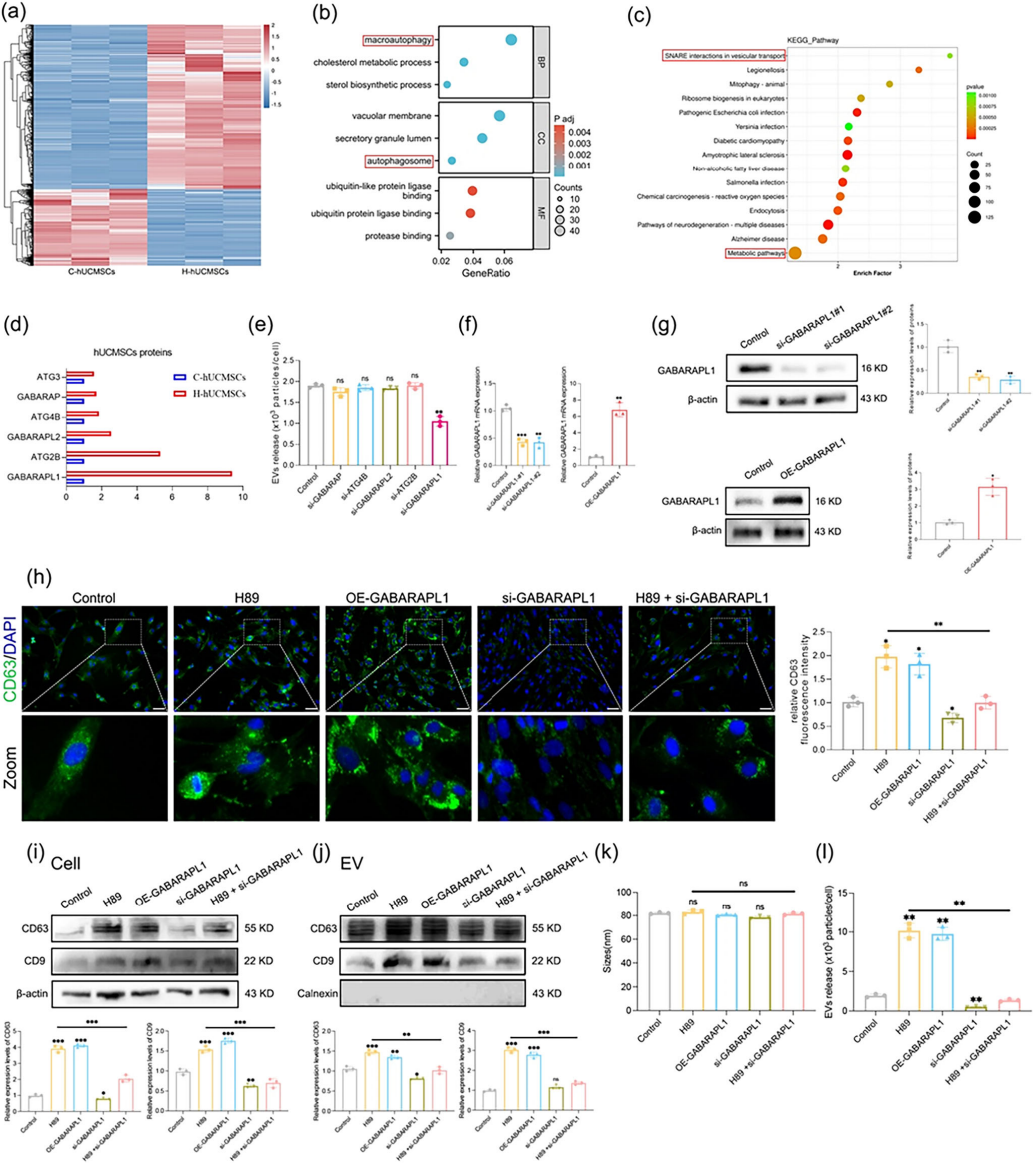
GABARAPL1 is associated with EV secretion and autophagy. (a) Cluster heatmap of differentially expressed proteins in H‐hUCMSCs versus C‐hUCMSCs. (b, c) GO and KEGG pathway enrichment analysis of differentially expressed proteins. (d) Fold changes in the expression of selected differentially expressed proteins linked to “macroautophagy”. (e) NanoFCM analysis of EV concentrations in hUCMSCs treated with siRNAs targeting genes encoding the selected proteins, with scramble siRNA as a control. (f, g) qPCR and WB validation of GABARAPL1 knockdown and overexpression efficiency. (h) hUCMSCs or GABARAPL1 knockdown hUCMSCs were pretreated with H89 for 48 h. PBS was added to hUCMSCs or hUCMSCs overexpressing GABARAPL1 as a control. Representative images of CD63 (green) were obtained through immunofluorescence staining (scale bar: 50 µm), and the relative fluorescence intensity was quantified. (i, j) The expression levels of CD63 and CD9 in cell lysates and EV fractions were assessed by WB, and relative protein levels were calculated. (k, l) NanoFCM analysis was performed to evaluate changes in EV size and concentration. All the data are presented as the mean ± SD (*n* = 3). Unpaired two‐tailed Student's t‐test and one‐way analysis of variance (ANOVA) were used to test for statistical significance. *p < 0.05, **p < 0.01, ***p < 0.001, ns: not significant.

To clarify the specific mechanism through which H89 pretreatment promotes EV secretion, we performed mass spectrometry analysis on H‐hUCMSCs (hUCMSCs pretreated with H89) and C‐hUCMSCs (hUCMSCs pretreated with PBS). We detected high expression of macroautophagy‐related proteins in the H‐hUCMSC group, and KEGG enrichment analysis revealed soluble N‐ethylmaleimide‐sensitive fusion factor attachment protein receptors (SNAREs) and metabolic pathways Figure 2a‐c. Six proteins were screened through GO analysis Figure 2d. We subsequently conducted a NanoFCM assay on the basis of the results of the siRNA experiments for these selected proteins Figure 2e. We used hUCMSCs treated with scramble siRNA as a negative control. On the basis of the selected siRNA results, we speculated that GABA type A receptor‐associated protein‐like 1 (GABARAPL1) is a reasonable candidate.

To assess whether H89‐mediated inhibition of GABARAPL1 affects EV formation, we validated the knockdown and overexpression efficiency of GABARAPL1 in hUCMSCs by qPCR and WB Figure 2f, g and collected the EVs secreted by these cells. The levels of CD63‐stained vesicles were increased in both the H89 and OE‐GABARAPL1 groups, whereas a significant decrease was noted in the si‐GABARAPL1 group Figure 2h. Furthermore, H89‐mediated GABARAPL1 overexpression increased the expression of CD63 and CD9 in cells, as well as the protein levels of EV components Figure 2i, j. NanoFCM analysis of nanoparticle size and distribution further supported the observation that EV production in the H89 group was notably reduced upon GABARAPL1 inhibition Figure 2k, l.

**FIGURE 3:**
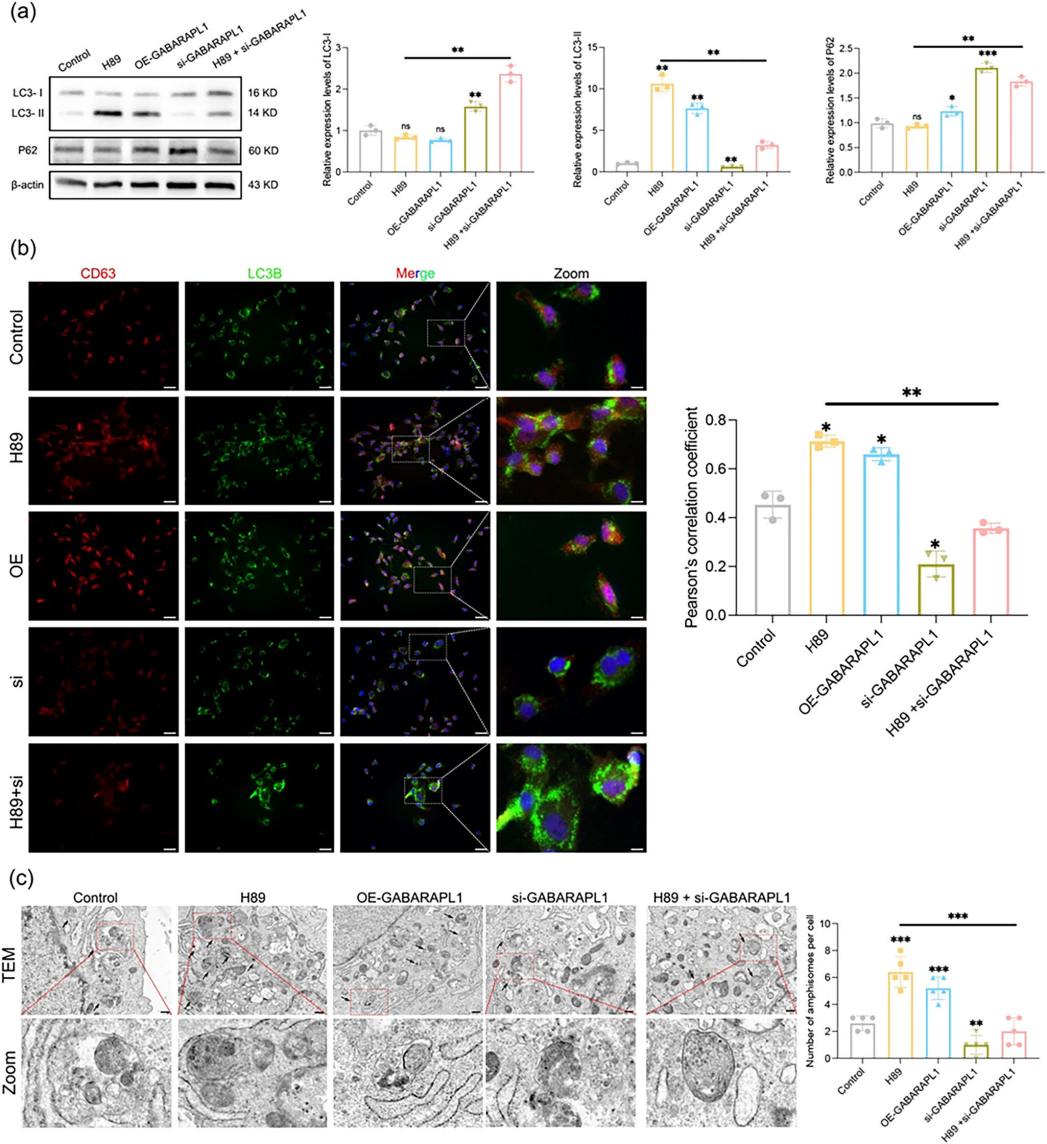
H89‐mediated GABARAPL1 promotes the formation of amphisomes and the secretion of EVs. (a) The expression of LC3 and p62 was measured by WB, and relative protein levels were calculated (*n* = 3). (b) Immunofluorescence staining was performed to visualize the MVB marker CD63 (red) and the autophagosome marker LC3B (green) (scale bar: 50 µm, *n* = 3). The correlation coefficient between the CD63 and LC3B signals was calculated. (c) Representative TEM images showing the number and morphology of amphisomes. The number of amphisomes per cell was quantified in random fields (*n* = 5). The black arrows indicate amphisomes (scale bar: 500 nm). All the data are presented as the mean ± SD. Unpaired two‐tailed Student's t test and one‐way analysis of variance (ANOVA) were used to test for statistical significance. *p < 0.05, **p < 0.01, ***p < 0.001, ns: not significant.

Autophagosomes and amphisomes are closely related structures involved in cellular autophagy and trafficking. Amphisomes can either fuse with lysosomes for degradation or with the plasma membrane to release their contents, including EVs, into the extracellular environment. GABARAPL1 is a member of the ATG8 family and plays a role in promoting the formation of hybrid organelles—amphisomes (MVBs and autophagosomes), thereby triggering the initiation of a non‐canonical autophagic metabolic pathway in cells (Ganesan and Cai 2021). The protein expression of LC3 and p62 was assessed to confirm the role of H89‐mediated GABARAPL1 in autophagy regulation Figure 3a. Immunofluorescence double‐staining revealed the colocalisation of LC3‐ and CD63‐positive MVBs, which served as an indicator of amphisome formation. Notably, we observed that treatment with H89 and GABARAPL1 increased the colocalisation of LC3 and CD63, suggesting increased generation of amphisomal structures Figure 3b. Additionally, transmission electron microscopy (TEM) revealed the accumulation of amphisome‐like structures in H89‐treated hUCMSCs Figure 3c.

### 2.3 H89 Alters EV Secretion Likely Through the SNARE‐Related Pathway

**FIGURE 4:**
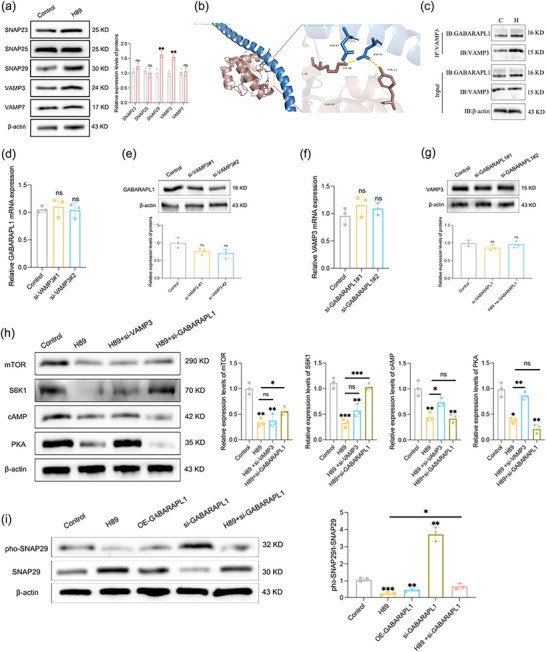
H89 inhibits the PKA–mTORC1 axis to promote EV secretion. (a) Western blotting was used to evaluate the expression levels of SNARE proteins (SNAP23, SNAP25, SNAP29, VAMP3, and VAMP7) in hUCMSCs treated with either the control or H89, and the relative expression levels were analyzed. (b) AlphaFold3 was used to predict the protein–protein interaction relationship between GABARAPL1 and VAMP3. (c) Coimmunoprecipitation (co‐IP) assays were performed to validate the interaction between GABARAPL1 and VAMP3, with IgG serving as the control. C denotes the control group; H denotes the H89‐treated group. (d, e) qPCR and WB were used to evaluate the mRNA and protein expression levels of GABARAPL1 after VAMP3 knockdown. (f, g) qPCR and WB were used to evaluate the mRNA and protein expression levels of VAMP3 after GABARAPL1 knockdown. (h) WB analysis of the regulatory effects of GABARAPL1 and VAMP3 on the mTORC1/S6K1 and cAMP/PKA signaling pathways. (i) WB analysis and quantitative results for phosphorylated SNAP29 (phospho‐SNAP29) and total SNAP29. All the data are presented as the mean ± SD (*n* = 3). Unpaired two‐tailed Student's t test and one‐way analysis of variance (ANOVA) were used to test for statistical significance. *p < 0.05, **p < 0.01, ***p < 0.001, ns: not significant.

MVBs or amphisomes fuse with the plasma membrane to facilitate the release of EVs, a process primarily mediated by SNARE proteins. In our previous study, KEGG pathway analysis revealed enrichment of the SNARE signaling pathway. Therefore, we examined the expression levels of several SNARE proteins, including SNAP23, SNAP25, SNAP29, VAMP3, and VAMP7. Compared with the control treatment, treatment with H89 significantly upregulated the expression of vesicle‐associated membrane protein 3 (VAMP3) and synaptosomal‐associated protein 29 (SNAP29) Figure 4a. VAMP3 and GABARAPL1 have been implicated in the regulation of autophagy. VAMP3 may contribute to autophagy through its role in vesicle fusion. Our results suggest that VAMP3 may interact with GABARAPL1, potentially facilitating the formation of amphisomes and thus promoting the secretion of EVs. We used AlphaFold3 to predict the interaction between VAMP3 and GABARAPL1 Figure 4b. Subsequently, we validated their interaction through a co‐IP assay, confirming that they can bind to each other Figure 4c. We then aimed to determine whether VAMP3 acts as an upstream or downstream regulator of GABARAPL1. To achieve this goal, we used qPCR and WB to assess the expression of GABARAPL1 after silencing VAMP3 using small interfering RNAs targeting the VAMP3 sequence. The results revealed that the downregulation of VAMP3 did not affect GABARAPL1 expression Figure 4d, e. Next, we examined the expression of VAMP3 following GABARAPL1 downregulation. The results revealed that the mRNA level of VAMP3 and its protein level remained unchanged Figure 4f, g. The above results revealed that VAMP3 is not a downstream effector protein of GABARAPL1. They are involved in different cellular biological processes but may be indirectly connected through certain overlaps in cellular functions.

H89, by modulating the mTORC1/S6K1/AKT and cAMP/PKA pathways (Grisan et al. 2021; Melick and Jewell 2020), can promote autophagy. To elucidate the specific molecular mechanisms by which GABARAPL1 and VAMP3 facilitate the formation of amphisomes, we investigated the effects of GABARAPL1 and VAMP3 on the protein expression levels of these two pathways. The results suggest that H89 can inhibit mTORC1/S6K1 signaling and PKA/cAMP signaling, while GABARAPL1 knockdown can rescue the inhibition of mTORC1, and VAMP3 knockdown can rescue the inhibition of PKA Figure 4h. These data suggest that H89 significantly affects the expression of SNAP29 and that the phosphorylation of SNAP29 influences the efficiency of EV release into the extracellular space. Therefore, we hypothesized that H89 promotes the fusion of amphisomes with the cell membrane by inhibiting SNAP29 phosphorylation. Next, we confirmed this hypothesis through Western blot assays Figure 4i. The findings suggest that H89 upregulates the expression of GABARAPL1, potentially regulating the mTORC1/S6K1 and PKA/cAMP pathways and thereby promoting the biological activity of GABARAPL1 and VAMP3. This, in turn, facilitates the fusion of amphisomes. Subsequently, dephosphorylated SNAP29 promotes the fusion of amphisomes with the cell membrane, thereby increasing the release of EVs into the extracellular space. Although the PKA inhibitor H89 was used to identify the role of this pathway in EV release, it has known off‐target effects on other kinases. To confirm the specific role of PKA, we performed genetic knockdown of its catalytic subunit, PRKACA Figure S1c. This knockdown robustly increased EV production, phenocopying the effect of H89 Figure S1d, e.

### 2.4 H‐EVs Promoted Liver Regeneration in Vivo and Hepatocyte Proliferation in Vitro

**FIGURE 5:**
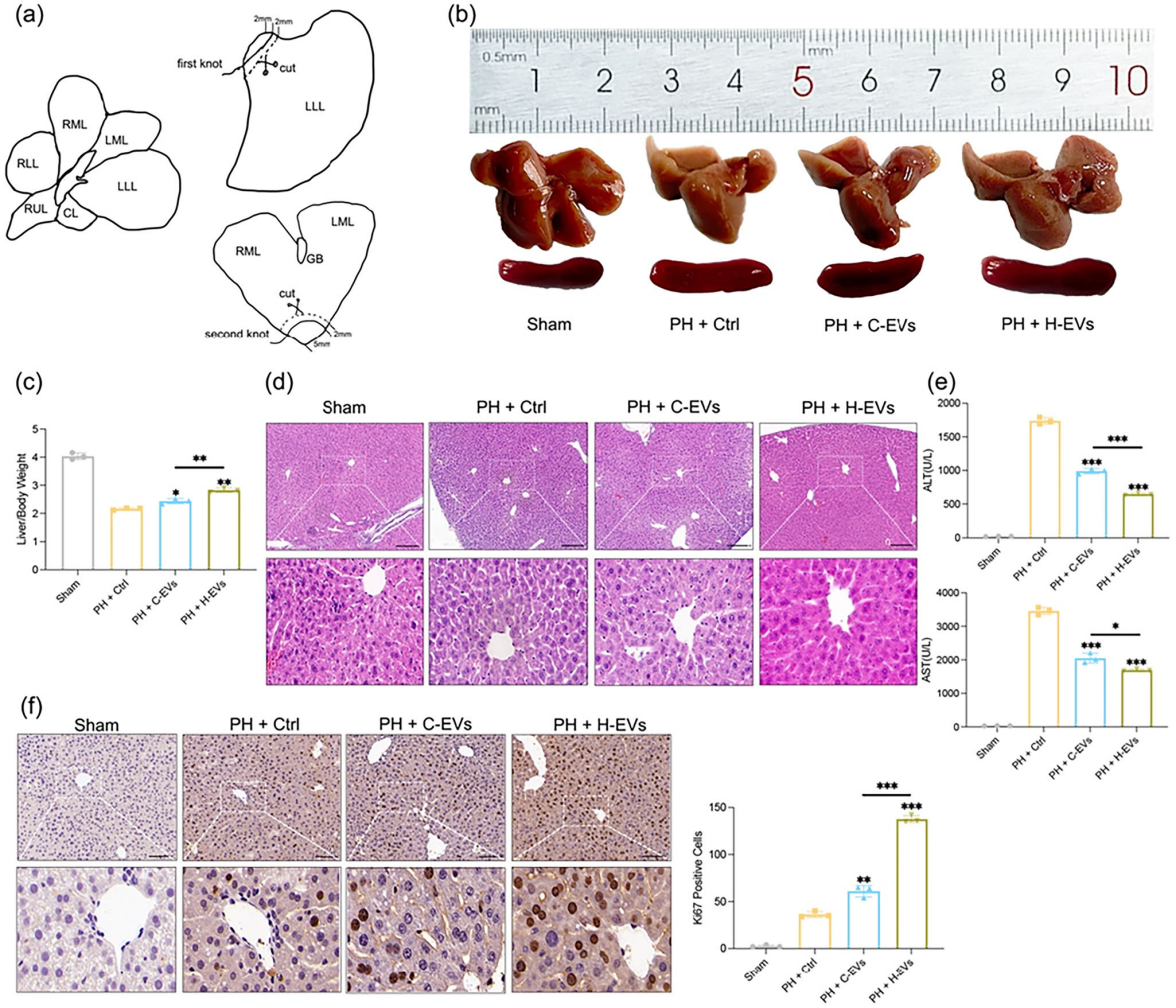
H89‐pretreated H‐EVs promote liver regeneration. (a) Schematic diagram of the 2/3 PH surgical model. Anatomical diagram of the liver: LLL, left lateral lobe; LML, left middle lobe; RML, right middle lobe; RUL, right upper lobe; RLL, right lower lobe; CL, caudate lobe; and GB, gallbladder. (b) Comparison of liver size changes in different treatment groups 36 h after 2/3 PH (the size of the liver in the image has been standardized for comparison). (c) Liver‐to‐body weight ratio measurements of the mice in the four groups at 36 h after PH. (d) H&E staining of liver tissues at 36 h after PH. Scale bar: 200 µm. (e) Serum levels of ALT and AST at 36 h after PH. (f) Ki67 immunohistochemical staining of liver tissue sections from mice at 36 h after PH. The positive cells were counted in random fields (*n* = 3). All the data are presented as the mean ± SD. Unpaired two‐tailed Student's t test and one‐way analysis of variance (ANOVA) were used to test for statistical significance. *p < 0.05, **p < 0.01, ***p < 0.001.

To further investigate whether H89 not only increases the secretion of EVs but also alters their biological activity, we compared the effects of EVs derived from H89‐pretreated hUCMSCs (H‐EVs) with those of control EVs (C‐EVs) on liver regeneration in the two‐third hepatectomy mouse model Figure 5a, as previously described(Mitchell and Willenbring 2008). The liver size and liver‐to‐body weight ratio were significantly greater in H‐EV‐pretreated mice than in control mice during the first 36 h posthepatectomy Figure 5b, c, S2, S4c. Additionally, H&E staining revealed a reduction in PH‐induced liver damage following EV administration, particularly in H‐EV‐treated mice Figure 5d, S3a, which was further supported by the decrease in serum ALT and AST levels Figure 5e, S3b, c. Hepatocyte proliferation, as assessed by Ki67 staining, was significantly enhanced in H‐EV‐treated mice at 36, 48, and 72 h after hepatectomy Figure 5f, S4. Taken together, our results suggest that H89 pretreatment alters the biological activity of hUCMSC‐EVs, thereby increasing their regenerative effects on the liver. Histopathological analysis of other organs in our animal model revealed no evident signs of toxicity or adverse morphological changes in the H‐EV treatment group compared with the control group Figure S7, serum biochemical analysis for markers of organ damage (e.g., markers of renal or cardiac injury) also revealed no significant increases Figure S8.

### 2.5 Changes in the Cargo Contents of EVs Derived From H89‐Pretreated MSCs

**FIGURE 6:**
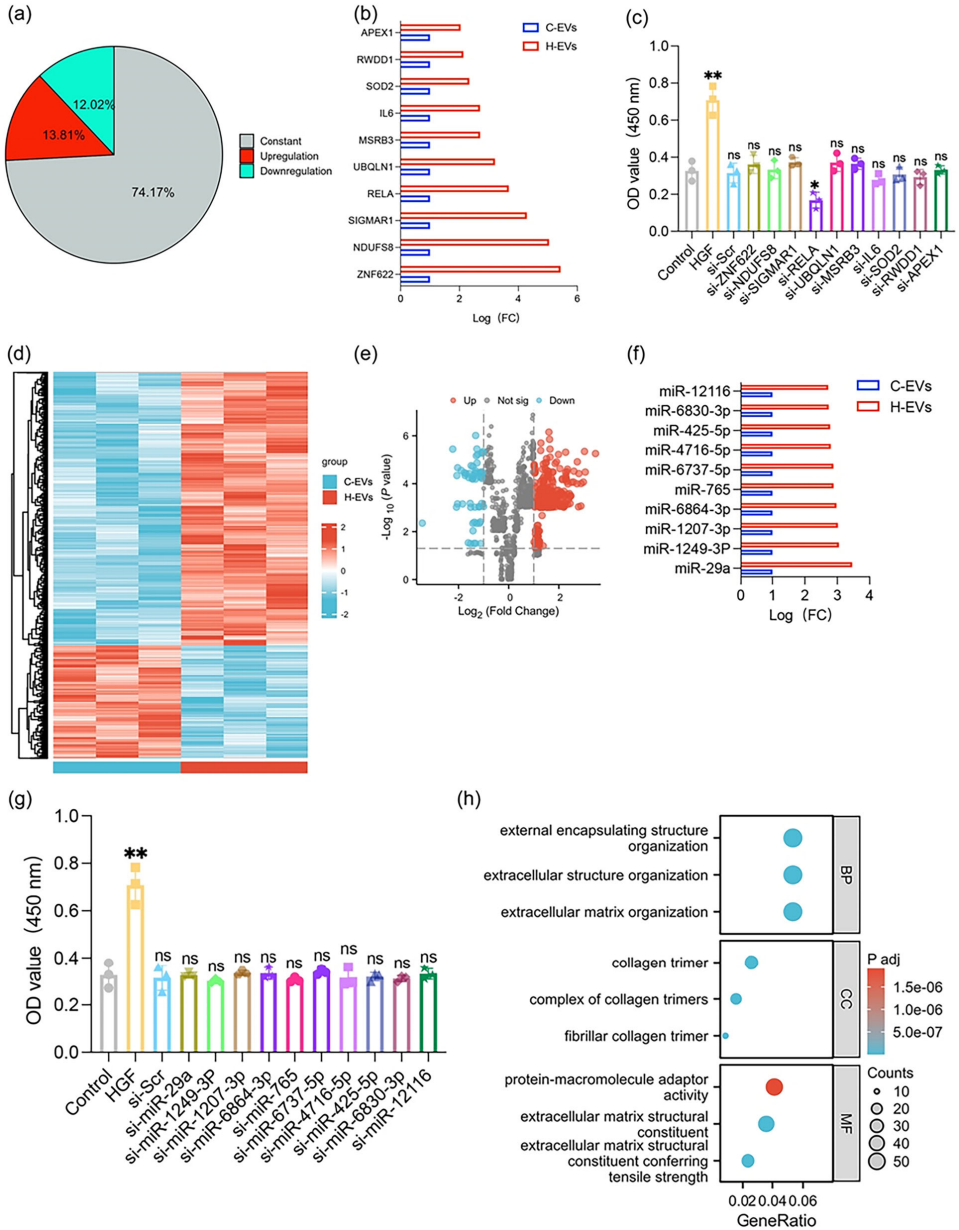
Modulation of the content of hUCMSC‐EVs by H89. (a) Proteomic analysis revealing the percentage of proteins that remained unchanged, were upregulated (>1.5‐fold), or were downregulated (<0.75‐fold) in H‐EVs compared with C‐EVs. (b) Selected differentially expressed proteins associated with “cell cycle regulation,” “energy metabolism,” and “oxidative stress,” along with their fold changes. (c) CCK‐8 assay demonstrating the effect of siRNA knockdown on AML12 cell proliferation. (d) Heatmap illustrating the changes in the expression of differentially expressed miRNAs between H‐EVs and C‐EVs. (e) Volcano plot highlighting the differentially expressed miRNAs. (f) The top 10 highly expressed miRNAs. (g) CCK‐8 assay to assess the effect of miRNA knockdown on AML12 cell proliferation. (h) GO and KEGG pathway analyses to identify the biological functions and pathways regulated by the target genes of miR‐29a. All the data are presented as the mean ± SD (*n* = 3). Unpaired two‐tailed Student's t test was used to test for statistical significance. *p < 0.05, **p < 0.01, ns: not significant.

Our in vivo tracking and relative clearance profiling demonstrated that compared with control treatment, H89 treatment did not alter the biodistribution or clearance of EVs, indicating that the enhanced biological activity of H‐EVs is primarily attributed to cargo modifications rather than changes in systemic trafficking Figure S5). To further analyse the regulatory effect of H89 on the bioactive components of hUCMSC‐EVs, proteomic mass spectrometry and miRNA sequencing were used to analyze the contents of H‐EVs and C‐EVs. We identified 4061 proteins in the EV proteome, among which 1049 were differentially expressed proteins Figure 6a. GO analysis of the differentially expressed proteins was then performed. To identify target proteins in H‐EVs, we analyzed proteins whose expression was upregulated > 4‐fold in H‐EVs versus C‐EVs (*P* < 0.05). Combining the results of the proteomic GO analysis with those of the molecular function analysis, 10 highly expressed proteins related to “cell cycle regulation”, “energy metabolism” and “oxidative stress” were selected Figure 6b. Next, we performed siRNA experiments on these proteins and screened RELA for significant regenerative effects on the basis of the CCK‐8 detection results for these selected proteins Figure 6c. As a transcription factor, RELA (NF‐kB p65 subunit) can directly target TNF‐a and IL‐6 in the nucleus to promote hepatic stellate cell (HSC) activation and is involved mainly in liver injury repair by regulating cell activation, inflammatory signaling, and extracellular matrix metabolism; however, persistent stimulation of RELA may cause excessive collagen deposition and lead to liver fibrosis (DeAngelis et al. 2001). Furthermore, on the basis of the integrated bioinformatic prediction of the miRTarBase and TargetScan databases, we screened the potential target genes of significantly different miRNAs from the miRNA sequencing results, and CCK‐8 assays revealed that they had no significant effect on the proliferation of AML12 cells Figure 6d‐g. However, among them, miR‐29a was significantly associated with extracellular matrix and collagen synthesis Figure 6h. A comprehensive literature analysis revealed that miR‐29a inhibits extracellular matrix and collagen synthesis (Matsumoto et al. 2016). On the basis of these findings, we hypothesized that RELA and miR‐29a in H‐EVs may promote liver regeneration by activating stellate cells while simultaneously suppressing collagen synthesis.

### 2.6 RELA/miR‐29a Interactome‐Mediated Reconfiguration of the Hepatic Stellate Cell Niche

**FIGURE 7:**
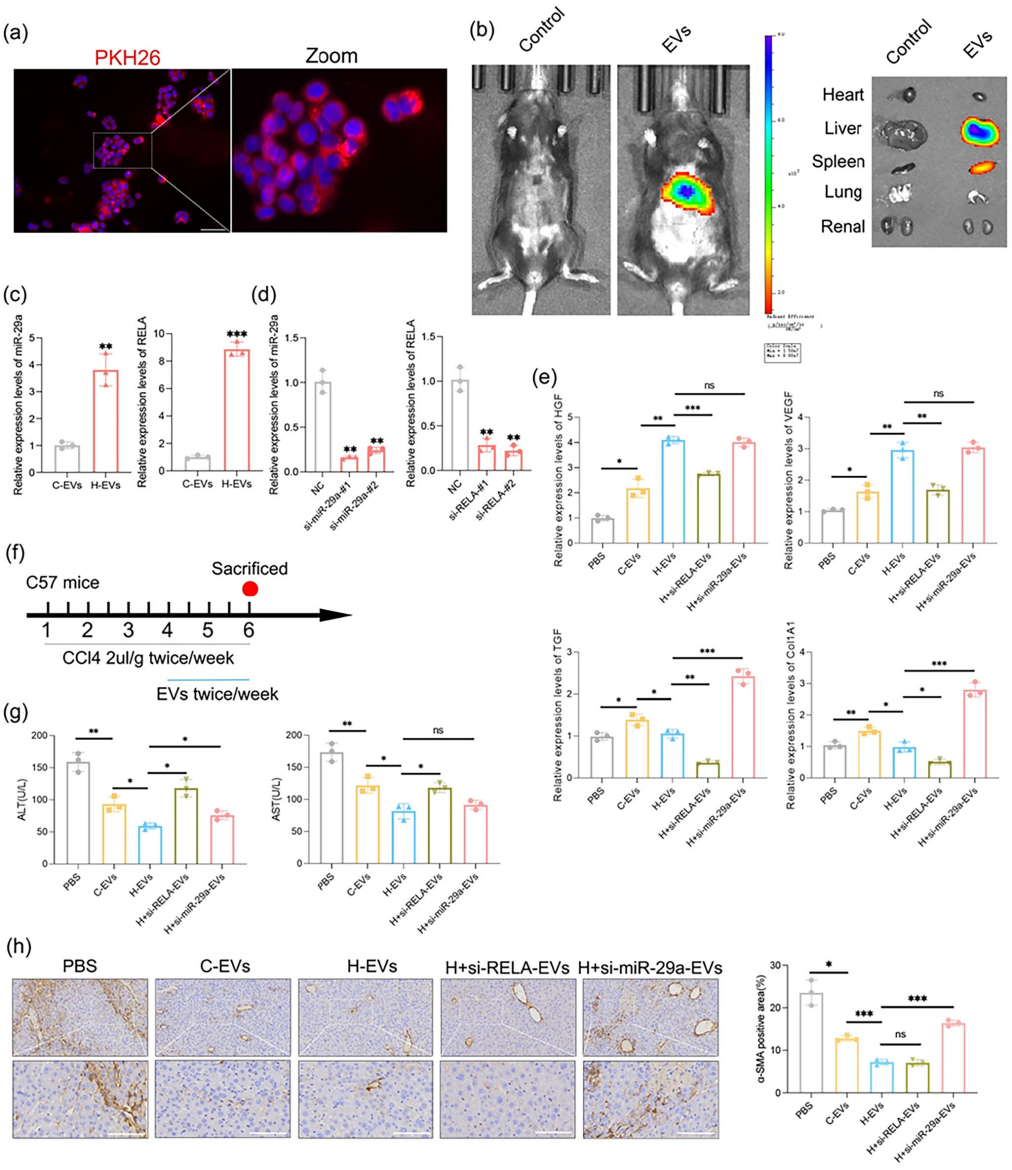
Regulation of hepatic stellate cell microenvironment homeostasis by RELA and miR‐29a derived from hUCMSC‐EVs. (a) In vitro fluorescence imaging of PKH26‐labeled EVs in LX‐2 cells. (b) In vivo fluorescence imaging of DiR‐labeled EVs. (c) qPCR analysis to detect the expression levels of miR‐29a and RELA in C‐EVs and H‐EVs. (d) qPCR analysis to assess the knockdown efficiency of miR‐29a and RELA in H‐EVs. (e) qPCR analysis to determine the relative expression levels of HGF, VEGF, TGF‐β, and Col1A1. (f) Construction of an EV‐based therapeutic model for chronic liver fibrosis in mice. (g) Serum levels of ALT and AST measured after treatment. (h) IHC staining for α‐SMA. All the data are presented as the mean ± SD (*n* = 3). Unpaired two‐tailed Student's t test and one‐way analysis of variance (ANOVA) were used to test for statistical significance. *p < 0.05, **p < 0.01, ***p < 0.001, ns: not significant.

**FIGURE 8:**
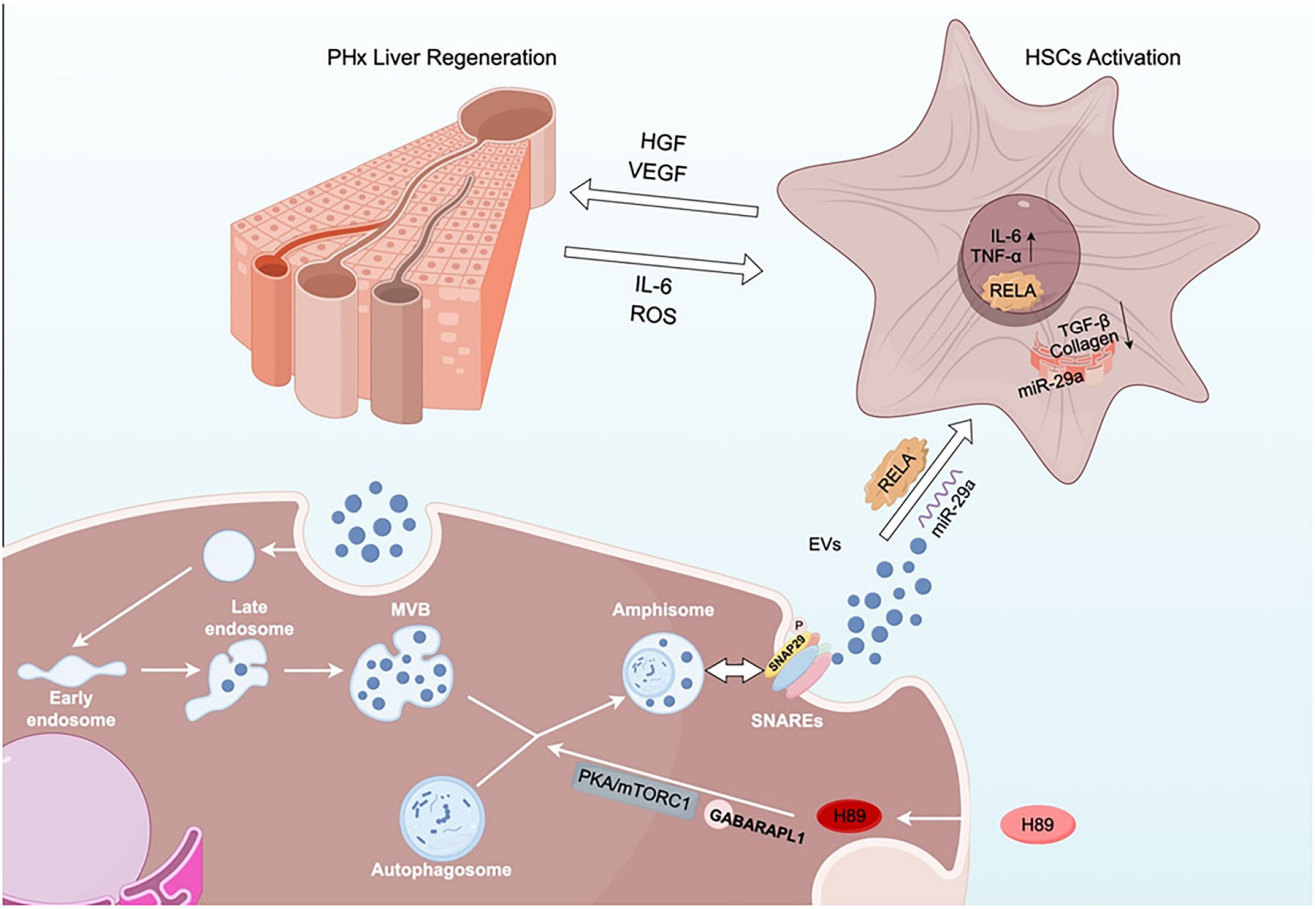
Schematic diagram of the mechanism by which H‐EVs lead to liver regeneration. H89 modulates the GABARAPL1/PKA/mTORC1 signaling axis in hUCMSCs, thereby promoting amphisome formation and enhancing EV secretion. H‐EVs regulate hepatic stellate cell (HSC) microenvironment homeostasis through a coordinated RELA/miR‐29a network, ultimately facilitating liver regeneration.

Next, LX‐2 cells were co‐cultured with hUCMSC‐EVs labeled with PKH26. Confocal microscopy analysis revealed that EVs were directly absorbed by cells Figure 7a. Similarly, after tail vein injection of DiR‐labeled EVs into mice for 24 h, we used IVIS imaging to assess the distribution of EVs, which were predominantly enriched in the liver and spleen Figure 7b, S5. Quantitative PCR demonstrated markedly higher levels of miR‐29a and RELA in H‐EVs than in C‐EVs Figure 7c. We subsequently downregulated miR‐29a and RELA in hUCMSCs and validated the knockdown efficiency using qPCR Figure 7d. Treatment of LX‐2 cells with C‐EVs, H‐EVs, H+si‐RELA‐EVs, and H+si‐miR‐29a‐EVs resulted in differential effects. qPCR revealed that H‐EVs significantly increased HGF, VEGF, and TGF‐β expression but downregulated Col1A1 and Col3A1 expression, and these effects were associated with the expression of RELA/miR‐29a in the EVs Figure 7e. We subsequently constructed a CCL4‐induced fibrosis mouse model Figure 7f. Four types of EVs were compared in terms of their therapeutic effects on the model mice. Our data revealed that si‐RELA‐EVs partially abolished the beneficial effects of H‐EVs on liver injury markers (AST and ALT), suggesting that RELA plays a crucial role in mediating hepatoprotective effects through EVs. In contrast, si‐miR‐29a‐EVs did not significantly affect AST or ALT levels Figure 7g. However, with respect to the expression of the fibrosis marker α‐SMA, si‐miR‐29a‐EVs partially reversed the increase in expression suppressed by H‐EVs, while si‐RELA‐EVs had a minimal affect Figure 7h. The effects of H+si‐RELA‐EVs and H+si‐miR‐29a‐EVs on liver regeneration were also confirmed in a two‐third hepatectomy mouse model. Compared with H‐EVs treatment, H+si‐RELA‐EVs treatment resulted in significant inhibition of liver regeneration. This was conclusively demonstrated by a significantly lower liver‐to‐body weight ratio and a marked reduction in hepatocyte proliferation (as measured by Ki‐67+ cells) in the H+si‐RELA‐EVs group Figure S6. These results directly confirm that the RELA component is indispensable for the enhanced regenerative function of H89‐primed EVs. Conversely, and unexpectedly, compared with H‐EVs treatment, H+si‐miR‐29a‐EVs treatment did not significantly promote liver regeneration. The regenerative capacity in this group remained robust and did not significantly differ from that in the group treated with standard H‐EVs. Moreover, histological and serum biochemical analyses conducted 4 weeks post‐injection confirmed that H‐EVs induced no detectable off‐target toxicity in major organs, further supporting their safety and therapeutic potential Figure S7, S8. These findings suggest that the synergistic regulatory mechanism of miRNAs and transcription factors may stimulate HSCs through RELA/miR‐29a, concurrently inhibiting collagen synthesis and regulating microenvironmental homeostasis to promote liver regeneration Figure 8.

## 3 Discussion

An increasing body of evidence suggests that EVs derived from human umbilical cord mesenchymal stem cells (hUCMSCs) can promote liver regeneration. However, the limited yield and instability of EVs present significant challenges for their clinical application. Mechanistic target of rapamycin complex 1 (mTORC1) is a pivotal signaling complex that regulates various cellular processes, including growth, metabolism, viability, and autophagy, primarily through the sensing of nutrient availability, oxygen, and energy levels (Li et al. 2020). Research has suggested that mTORC1 activation may inhibit autophagy and suppress EV secretion (Bhaoighill et al. 2023; Zou et al. 2019). Thus, inhibiting mTORC1 activity may represent a potential strategy to increase EV secretion.

In the present study, we showed that H89, a selective protein kinase A (PKA) inhibitor (Bedioune et al. 2024), significantly promotes the secretion of hUCMSC‐derived EVs by inhibiting the mTORC1 pathway. The small molecular nature of H89 allows for rapid cellular uptake and tissue penetration, while also offering advantages in terms of manufacturing scalability and quality control compared to complex biological ligands. Although the PKA inhibitor H89 was used to identify the role of this pathway in EV release, it has known off‐target effects on other kinases. To confirm the specific role of PKA, we performed genetic knockdown of its catalytic subunit, PRKACA. This knockdown robustly increased EV production, phenocopying the effect of H89. This genetic evidence, combined with the congruent suppression of mTORC1 signaling, strongly supports the conclusion that PKA inhibition is the primary mechanism responsible for enhanced EV release. While a contribution from off‐target effects cannot be completely ruled out, the consistency of the data across pharmacological and genetic approaches confirms the central role of the PKA‐mTORC1 axis.

Our results indicate that H89 not only enhances hUCMSC‐EV secretion but also has similar effects on other cell lines, suggesting that this effect may have broad applicability. Notably, while H89 treatment did not significantly alter the size or morphology of the hUCMSC‐EVs, it substantially increased their concentration. These findings provide novel insights into the regulation of EV secretion and offer potential strategies for the clinical application of EVs as therapeutic carriers. In addition, our comprehensive in vivo tracking and relative clearance profiling demonstrated that both control EVs (C‐EVs) and H89‐treated EVs (H‐EVs) exhibit comparable biodistribution, organ uptake, and blood clearance profiles. It is important to acknowledge the inherent limitations of current in vivo EV tracking methodologies. Lipophilic dyes such as DiR may generate false‐positive signals following EV degradation due to dye redistribution to tissues or lipoproteins. In addition, our systemic clearance assessment relied on quantifying CD63‐positive particles. Because CD63 represents only a subset of heterogeneous EVs populations, this approach substantially underestimates total circulating EV concentrations. Notably, there is currently no universally accepted gold standard for determining the absolute in vivo pharmacokinetics of EVs. Therefore, our data should not be interpreted as absolute pharmacokinetic measurements, but rather as a comparison of relative clearance trends between C‐EVs and H‐EVs under identical experimental conditions. These findings indicate that the enhanced biological activity of H‐EVs observed in functional assays arises primarily from alterations in their molecular cargo rather than differences in their in vivo trafficking behavior, thereby supporting their continued therapeutic potential. Our biodistribution data show significant accumulation of H‐EVs in the liver and spleen. This natural tropism toward the mononuclear phagocyte system (MPS) is beneficial for liver‐targeted therapies, as it ensures a high local concentration of regenerative cargo in hepatectomy models. However, this rapid clearance by the liver and spleen presents a significant challenge for targeting non‐MPS tissues (e.g., heart or brain), and would require additional surface engineering to prevent hepatic trapping.

Compared with other major strategies, H89 demonstrates significant advantages in enhancing extracellular vesicle (EV) secretion. Physical stimuli (e.g., hypoxia (Gupta et al. 2022) or LED light irradiation (Ruan et al. 2022)), which are typically used to increase EV yield by mimicking physiological stress, often activate a broad and complex cellular stress response, leading to batch‐to‐batch variability and unpredictable changes in EV cargo. In contrast, H89 acts through a more specific pharmacological inhibition of PKA, resulting in a more reproducible and controlled alteration of the EV biogenesis pathway, which likely contributes to the consistent functional enhancement observed in H‐EVs. Compared with genetic engineering approaches (e.g., overexpression of HIF‐1α (Sun et al. 2020) or Shp2 (Zhang et al. 2021)), which can achieve massive increases in EV secretion but face challenges in clinical translation because of safety concerns with permanent genetic modifications and viral vectors, H89 offers a non‐genetic, small‐molecule solution. The stimulation by H89 is transient, occurring only during the preconditioning phase, thus presenting a lower regulatory hurdle for future therapeutic applications. Furthermore, compared with other chemical/pharmacological inducers (e.g., INF‐γ (Serejo et al. 2019), or LPS (Li et al. 2021)), which are potent but often cytotoxic at effective concentrations, H89 treatment at optimized concentrations effectively enhances EV secretion without causing significant cytotoxicity, making it a more “cell‐friendly” alternative.

GABA type A receptor‐associated protein like 1 (GABARAPL1) is a protein related to the γ‐aminobutyric acid type A receptor (GABAAR), which belongs to the GABARAP family. It shares a high degree of homology with other proteins in this family, such as GABARAP and GABARAPL2 (Chakrama et al. 2010). GABARAPL1 plays a crucial role in the process of autophagy, particularly in the formation, maturation, and degradation of autophagosomes (Zheng et al. 2023a), and it interacts with autophagy‐related proteins such as LC3, Atg4B, and Atg7, contributing to the expansion and closure of autophagosomes and facilitating the degradation and recycling of the contents of autophagosomes (Agrotis et al. 2019). Vesicle‐associated membrane protein 3 (VAMP3), also known as cell pressure‐sensitive VAMP (VAMP/synaptobrevin III), is a widely expressed v‐SNARE protein involved in both exocytosis and endocytosis processes (Coelho et al. 2022). Research has indicated that VAMP3 also participates in the fusion of MVBs with the plasma membrane (Kumar et al. 2020). In the formation of amphisomes, VAMP3 facilitates the alignment and fusion of the autophagosome membrane with the lysosomal membrane through its interaction with t‐SNAREs (Moreau et al. 2013). VAMP3 also promotes the formation of amphisomes during the fusion of MVBs and autophagosomes (Fader et al. 2009).

In this study, we explored how H89 promotes the secretion of hUCMSC‐derived extracellular vesicles (hUCMSC‐EVs) by regulating the expression of GABARAPL1. We also investigated the key role of the interaction between GABARAPL1 and VAMP3 in the formation of amphisomes and the release of hUCMSC‐EVs. As an important member of the ATG8 family, GABARAPL1 plays a crucial role in the formation and maturation of autophagosomes. Our findings revealed that H89 upregulates the expression of GABARAPL1 while inhibiting the mTORC1/S6K1/AKT and cAMP/PKA signaling pathways. This regulatory mechanism likely promotes the formation and maturation of amphisomes through a non‐classical autophagy pathway, thereby enhancing the secretion efficiency of hUCMSC‐EVs. As a key structure in the autophagy process, the amphisome effectively facilitates the release of the contents of hUCMSC‐EVs into the extracellular environment. Previous studies have suggested that SNARE complexes assist in the fusion of autophagosomes with lysosomes, thereby facilitating the progression of autophagy (Tian et al. 2021). SNAP29, a member of the SNAP protein family, mainly regulates the fusion process of vesicles by participating in the disassembly and reassembly of SNARE complexes (Pellegrini et al. 2023). Our results suggest that H89 may regulate the role of SNAP29 in the disassembly and reassembly of the SNARE complex by inhibiting its phosphorylation, thus promoting the fusion of amphisomes with the cell membrane. On the basis of these findings, we propose a novel regulatory mechanism for EV secretion. H89 enhances the formation and maturation of amphisomes by promoting the expression of GABARAPL1 and VAMP3. H89 also facilitates plasma membrane fusion through the GABARAPL1‐mediated dephosphorylation of SNAP29, thereby promoting the secretion of hUCMSC‐EVs. This mechanism not only reveals the potential role of H89 in regulating autophagy and EV secretion but also provides important insights into the new functions of autophagy pathways in cell communication. Furthermore, because H89 specifically unlocks the amphisome‐mediated secretion pathway, it likely influences the subtype distribution of the secretome, enriching the yield of MVB‐derived small EVs (exosomes) over plasma membrane‐derived microvesicles. This pathway‐specific enhancement is a key factor contributing to the uniquely altered and optimized therapeutic cargo profile observed in H‐EVs.

The liver possesses a remarkable regenerative capacity, primarily mediated by the proliferation and differentiation of hepatocytes following partial hepatectomy or injury (Yagi et al. 2020). However, this regenerative response is often impaired by several pathological factors, including oxidative stress, apoptosis, and chronic inflammation (Michalopoulos and Bhushan 2021; Wang et al. 2024). In this study, we investigated the therapeutic potential of EVs derived from H89‐pretreated hUCMSCs (H‐EVs) in the context of liver injury and regeneration. Our findings demonstrate that H‐EVs markedly enhanced liver regeneration and significantly attenuated posthepatectomy injury, likely through their distinct biological activities. In addition, our short‐term (4 weeks) toxicology data demonstrate that H‐EVs maintain a favorable safety profile. However, it is important to acknowledge that this represents a preliminary, short‐term assessment. For future clinical translation, comprehensive, long‐term, GLP‐compliant toxicology and pharmacokinetics studies will be strictly required to fully evaluate the safety profile of H‐EVs.

To gain deeper insights into the biological mechanisms of H‐EVs, we performed a comprehensive omic analysis of their contents. Through miRNA sequencing and proteomic mass spectrometry, we compared the molecular compositions of H89‐pretreated (H‐EVs) and control (C‐EVs) EVs. Our results revealed that H‐EVs are enriched in proteins associated with “cell cycle regulation,” “energy metabolism,” and “oxidative stress,” which is likely involved in hepatocyte proliferation, repair, and regeneration. Functional analysis further revealed RELA as one of the most highly expressed proteins. RELA is a subunit of nuclear factor kappa B (NF‐κB), a critical regulator of inflammatory responses, immune modulation, and cell survival (Moriya et al. 2023; Zhang et al. 2024). Previous studies have shown that excessive activation of RELA can contribute to liver fibrosis and chronic liver diseases, whereas moderate RELA activation may facilitate liver regeneration and repair (Boya et al. 2001; Wang et al. 2019). During hepatic stellate cell (HSC) activation and liver fibrosis progression, RELA significantly enhances HSC activation and proliferation by regulating the expression of multiple inflammation‐ and fibrosis‐related genes, including pro‐inflammatory cytokines (Gehrke et al. 2018; Geisler et al. 2007) (e.g., IL‐6 and TNF‐α) and growth factors (Enguita et al. 2019; Fan et al. 2022; Yang et al. 2008) (e.g., TGF‐β, HGF, and VEGF). Through siRNA‐mediated knockdown, we demonstrated that RELA plays a pivotal role in promoting liver regeneration. Thus, RELA appears to regulate the inflammatory response following liver injury, contributing to liver repair and regeneration.

In addition to proteins, EVs are enriched with various miRNAs, which regulate liver regeneration by modulating the expression of target genes (Matsuzaka and Yashiro 2022). For example, miR‐190b‐5p and miR‐296‐3p, two novel antifibrotic miRNAs, significantly inhibit liver fibrosis progression by targeting HAS2 and ITGA6 (Markovic et al. 2025). miR‐106b‐5p promotes liver regeneration by targeting vimentin (Lu et al. 2020). In our study, we observed that miR‐29a was highly expressed in H‐EVs and closely associated with the extracellular matrix and collagen synthesis. miR‐29a plays a key role in suppressing collagen synthesis, thereby inhibiting liver fibrosis and regulating the liver microenvironment (Huang et al. 2018; Xue et al. 2024). Our findings suggest that RELA and miR‐29a cooperatively regulate hepatic stellate cells to maintain liver microenvironment homeostasis. RELA facilitates liver repair by activating HSCs to secrete HGF and VEGF, while miR‐29a mitigates liver fibrosis by inhibiting collagen synthesis. This synergistic regulatory mechanism effectively modulates the liver microenvironment, promoting liver regeneration and repair.

These findings elucidate, for the first time, the pivotal role of H89 pretreatment in EV‐based therapy through a “dose–effect synergy” strategy. This approach not only significantly enhances EV yield but also augments the functional activity of EVs, providing a novel theoretical foundation and technical framework for engineered EV‐based liver regeneration therapies.

Compared with genetic engineering, the H89‐based approach offers significant advantages for clinical EV production, including scalability compatible with cGMP bioreactors and reduced complexity. While challenges such as serum‐free media optimization and large‐scale purification remain, H89 treatment does not exacerbate these challenges. Regulatory benefits include precise control over H89 dosage, leading to more consistent products. The main safety concern—residual H89—can be addressed through robust purification and quality control. Functional testing confirms enhanced EV efficacy, and the approach represents a viable process change for clinical cell types, improving product quality without compromising safety.

However, this study has certain limitations. First, the molecular interactions among proteins during EV therapy have not been thoroughly investigated, which limits the understanding of the specific mechanisms underlying H89 pretreatment. Second, in terms of the spatiotemporal dynamics of liver regeneration, only preliminary observations and analyses were conducted in this study, and a comprehensive dynamic regulatory network was not provided. These shortcomings offer directions and opportunities for improvement in future research.

## 4 MATERIALS AND METHODS

### 4.1 Cell Culture

Human umbilical cord mesenchymal stem cells were prepared previously. In this study, third‐passage human umbilical cord‐derived mesenchymal stem cells (hUCMSCs) were cultured in AM‐V serum‐free medium (Tbdscience, Tianjin, and China). The murine hepatocyte cell line AML12 was purchased from Procell Life Science & Technology Co., Ltd. (Wuhan, China). These cells were cultured in DMEM/F12 supplemented with 10% fetal bovine serum (FBS; Gibco, USA) and 1% ITS (insulin, transferrin, and selenium; Cyagen, USA). The human hepatic stellate cell line LX2 was purchased from iCell Bioscience, Inc. (Shanghai, China). These cells were cultured in DMEM medium supplemented with 10% FBS. All cells were incubated at 37°C in 5% CO2.

### 4.2 Cell Viability Assessment

Cell viability was analyzed by Cell Counting Kit‐8 (CCK‐8; APExBIO, USA) assay according to the manufacturer's protocols. Cells were seeded and cultured at a density of 8 × 10^3^/well in 96‐well microplates (Corning, USA). After treatment for 24 h, 10 µL of CCK‐8 reagent was added to each well, and the cells were then cultured for 3 h. All experiments were performed in triplicate. The absorbance was analyzed at 450 nm using a microplate reader (Thermo Scientific, USA).

### 4.3 Extracellular Vesicle Isolation

Until the cells reached 70%–80% confluence, the medium was replaced with fresh AM‐V serum‐free medium, and the MSCs were cultured for another 48 h. By replacing the spent medium with fresh medium at a specific, standardized confluence (70%–80%), we ensured that all the cells in our experiments were provided with an identical, nutrient‐replete environment for the subsequent 48‐hour EV production period. The supernatant was subsequently collected and centrifuged at 300 × g for 5 min at 4°C to pellet the residual cells, which were subsequently centrifuged at 2000 × g for 10 min at 4°C to remove cellular debris. Then, the samples were transferred to ultracentrifuge tubes and centrifuged at 10,000 × g for 30 min at 4°C to remove the mass vesicles. The EVs were harvested by ultracentrifugation twice at 100,000 × g for 2 h at 4°C. After the supernatant was removed, the EVs were resuspended in 100 µL of PBS and stored at −80°C.

### 4.4 Transmission Electron Microscopy (TEM)

Two microliters of diluted EVs were dropped onto a copper electron microscopy grid, blotted, negatively stained with 2% aqueous phosphotungstic acid for 2 min, rinsed with PBS 3 times, blotted, and finally imaged with an H7500 transmission electron microscope at 80 kV. For each EV sample, grids were prepared in triplicate. From each grid, images were systematically captured from five randomly selected fields of view at 12,000x magnification that contained a well‐distributed, nonaggregated population of vesicles.

### 4.5 Nanoflow Cytometry (NanoFCM)

To determine the concentration and particle size, a NanoFCM N30E was used according to the manufacturer's protocol. The instrument was tested and adjusted with standard QC beads, and then properly diluted samples were loaded and recorded for 1 min. The particle concentration and size were calculated with a calibration curve.

### 4.6 Western Blot Analysis

Cells were lysed in RIPA lysis buffer containing PMSF (Beyotime Biotechnology, China), after which the protein concentration was quantified by BCA assay (Beyotime Biotechnology, China). Additionally, EVs were directly lysed in 2× SDS loading buffer. The samples were boiled and then separated by sodium dodecyl sulfate–polyacrylamide gel electrophoresis and transferred onto a PVDF membrane (Millipore, USA). After being blocked in 5% milk for 1 h, membranes were incubated with the indicated primary antibodies Table S1, followed by incubation with horseradish peroxidase (HRP)‐conjugated secondary antibodies. The signals were visualized with enhanced chemiluminescence (ECL) reagent (YEASEN, China). To assess the protein levels, the density of the bands in three independent experiments was quantified by ImageJ (NIH).

### 4.7 Immunofluorescence Analysis

For the immunofluorescence analysis, we used a slightly modified version of a previously used immunofluorescence analysis method. In brief, cells adhered to 24‐well plates were washed with phosphate‐buffered saline (PBS), fixed with 4% paraformaldehyde, and permeabilized with 1% Triton X‐100. The cells were blocked in QuickBlock Immunostaining Blocking Solution (Beyotime Biotechnology, China) and reacted with antibodies as indicated. The primary antibodies used are listed in the supplemental materials. Alexa Fluor‐conjugated secondary antibodies (1:200; Beyotime Biotechnology, China) were used with fluorophores excitable at wavelengths of 488, 594, or 647 nm. The nuclei were counterstained with DAPI (Life Sciences, USA). Images were captured using a confocal laser microscope (Nikon, Tokyo, Japan). Exposure to light was strictly avoided throughout the entire procedure.

### 4.8 Immunoprecipitation

hUCMSCs were cultured in 6‐well plates at 37°C with 5% CO2. After 24 h, the experimental group was treated with 10 µM H89 for 24 h, while the control group received PBS. At > 80% confluence, the cells were lysed with 600 µL of prechilled lysis buffer per well, scraped, and transferred to 1.5 mL tubes. Cells were disrupted using 30% power ultrasound (10–20‐s pulses) with cooling to prevent protein degradation. The homogenate was centrifuged at 13,000 rpm and 4°C for 15 min to separate the supernatant. Protein A+G magnetic beads were subsequently washed three times with 1 mL of lysis buffer (3000 rpm, 1 min). For immunoprecipitation, 5 µL of washed beads was mixed with 5 µL of target protein antibodies in IP tubes, with controls receiving IgG antibodies. Input samples were prepared with 2× SDS buffer. The supernatant was added to IP tubes, mixed, and incubated at 4°C for 12–16 h. After incubation, the contents of the IP tubes were washed three times with 1 mL of lysis buffer, and the beads were boiled in 1× SDS buffer for 5 min to collect IP samples. IP and input samples were separated by SDS–PAGE, transferred to membranes, and probed with target protein and phosphorylation‐specific antibodies. Protein expression and phosphorylation levels were analyzed after development.

### 4.9 Quantitative Real‐Time Polymerase Chain Reaction (qRT–PCR)

Total RNA from cultured cells was extracted using TRIzol reagent (Invitrogen) according to the manufacturer's instructions. Quantitative real‐time PCR (qRT–PCR) was performed (Bio‐Rad, Hercules, CA, USA), and the sequences of the primers used for the SYBR green PCR system are shown in the supplemental materials Tables S2, S3. All the experiments were performed in triplicate.

### 4.10 Mass Spectrometry Proteomics and Data Analysis

hUCMSCs were cultured in T182 flasks until they reached 70% confluence and were then pretreated with 10 µM H89 for 48 h (with PBS as a control); subsequently, EVs were enriched from the supernatant via ultracentrifugation. For protein quantification, the samples were lysed in buffer (7 M urea, 2% SDS, and protease inhibitors), sonicated (2 s on/5 s off, 1 min) on ice, and lysed for 2 h. The lysates were subsequently centrifuged at 13,000 rpm (4°C, 20 min), after which the supernatant was recentrifuged. The protein concentration was determined by BCA assay. Proteins were digested with trypsin into peptides, purified via solid‐phase extraction to remove impurities, and analyzed by LC–MS/MS. Peptides were separated by liquid chromatography and detected on the basis of the mass‐to‐charge ratio (m/z). MS/MS enhanced identification accuracy. Data were processed using Mascot, Proteome Discoverer, or MaxQuant, and the spectra were compared to protein databases for identification and quantification (label‐free or labeled methods). Bioinformatic analysis revealed annotated protein functions and cellular localization.

### 4.11 High‐Throughput Sequencing

The QIAseq miRNA Library Kit (Qiagen, USA) was used to determine the miRNA expression profiles. Total RNA (2 µL) was extracted with TRIzol, and the RNA purity and concentration (100–150 ng/µL) were determined. An Illumina sequencer (HiSeq^TM^ 2500) was used after passing a quality inspection. miRNAs were subsequently confirmed and statistically analyzed.

### 4.12 Animal Experiments

The experimental protocol involved 6–8‐week‐old mice (weighing 25 ± 1.5 g) randomly divided into four groups (*n* = 25 per group) using a random number table method: two EV treatment groups (C‐EVs and H‐EVs) and two control groups (Ctr and Sham). Animals in the EV treatment groups received an intravenous tail injection of 1×10^9^ EVs (200 µL) 2 h before surgery, whereas those in the control groups were injected with an equal volume of sterile phosphate‐buffered saline (PBS). A 70% liver resection model was established using the Mitchell and Willenbring method (Mitchell and Willenbring 2008); the mice were fasted for 12 h prior to surgery but allowed free access to water. Anesthesia was induced with isoflurane via continuous inhalation. The surgical procedure included skin preparation and disinfection using a depilatory razor and iodophor; a midline abdominal incision to expose the liver, with a 5 mL syringe placed under the mouse's lumbar region to facilitate exposure of the left lateral and middle liver lobes; ligation and resection of the hepatic pedicle using 4–0 sutures, followed by removal of the left lateral and middle lobes; and precise hemostasis with gauze and layered suturing to ensure no postoperative leakage.

### 4.13 Labeling and Absorption of EVs

Two fluorescent labeling methods were used to track EVs: PKH26 and DiR. For PKH26 labeling, EVs were quantified using a BCA protein kit to construct a standard curve. A 100 µM PKH26 working solution was prepared by diluting the stock with Diluent C in the dark. The solution was added to the EVs, which were incubated at room temperature for 10 min, and the reaction was terminated with sterile PBS. After ultracentrifugation, the pellet was resuspended in PBS and stored at 4^°^C. For cellular experiments, LX‐2 cells were seeded at 5×10^4^ cells/cm^2^, incubated with labeled EVs (100 µg/mL) for 6 h, washed, fixed, and stained with DAPI. Confocal microscopy images were acquired at 543 nm (PKH26) and 405 nm (DAPI), and the fluorescence intensity was analyzed using ImageJ. For DiR labeling, a 100 µM DiR working solution was prepared with prechilled PBS. The solution was incubated with EVs at 37°C for 30 min, terminated with PBS, and then purified by ultracentrifugation. The labeled EVs were resuspended in PBS and stored at 4°C. In vivo imaging was performed on 6–8‐week‐old C57 mice. Mice in the experimental group received 1×10^9^ labeled EVs via tail vein injection, whereas those in the control group received PBS. Fluorescent live imaging with an IVIS system was performed at different time points, and the fluorescence intensity was quantified using Living Image software.

### 4.14 Histological Analysis

Liver samples were fixed with 4% paraformaldehyde, dehydrated, embedded in paraffin, sliced into 4‐µm sections and stained with H&E. The samples were then observed under a microscope to detect liver injury.

### 4.15 Production of siRNA‐Modified EVs

Human umbilical cord mesenchymal stem cells (MSCs) were seeded in T175 culture flasks and grown in 35 mL of AM‐V serum‐free medium until they reached 60%–70% confluence. Small interfering RNAs (siRNAs) targeting RELA or miR‐29a were synthesized by Tsingke Biotechnology (Guangzhou, China). Lipofectamine 3000 (Invitrogen, USA) was used for transfection according to the manufacturer's instructions. Specifically, for each T175 flask, 1382 pmol of siRNA (yielding a final concentration of approximately 39.5 nM) and 138 µL of Lipofectamine 3000 were added to the 35 mL culture system. 48 h later, the transfection efficacy of the mRNAs was verified by qPCR. The protein level of RELA was determined by Western blotting after 72 h. The siRNA sequences used in this study are listed in Supplementary Table S3. The transfected cells were incubated for an additional 48 h to allow maximal gene knockdown and subsequent secretion of engineered EVs into the conditioned medium. The conditioned medium was collected and centrifuged to remove cells and debris. H+si‐RELA‐EVs, H+si‐miR‐29a‐EVs, and control H+si‐NC‐EVs were then isolated by ultracentrifugation, as described in “Section 4.3 Extracellular vesicle isolation”.

### 4.16 Statistical Analysis

All the data are presented as the mean ± SD. Unpaired two‐tailed Student's t test and one‐way analysis of variance (ANOVA) were used to test for statistical significance. *P* < 0.05 was considered to indicate a statistically significant difference. All the statistical analyses were performed using SPSS (version 21.0) and GraphPad Prism 9.

#### AUTHOR CONTRIBUTIONS

Yu Fu: writing – original draft, writing – review and editing, conceptualization, methodology. Yi Ma: methodology, investigation. Jiajun Zhang: methodology, investigation. Liwei Liang: investigation. Ting Li: investigation. Zeyi Guo: investigation. Zhongzhe Li: investigation. Lei Feng: investigation, funding acquisition. Yi Wang: investigation. Guolin He: formal analysis. Shao Li: formal analysis. Yang Li: formal analysis. Xiaoping Xu: writing – original draft, writing – review and editing, formal analysis. Hui Liao: formal analysis, writing – review and editing, writing – original draft. Yi Gao: formal analysis, writing – original draft, writing – review and editing, funding acquisition.

## Conflicts of Interest

The authors declare no conflicts of interest.

## Supporting information




**Supporting Information**: jev270285‐Sup‐0001‐SuppMat.docx

## Data Availability

The data that support the findings of this study are available from the corresponding author upon reasonable request.
