# Supplementary material for: The Small Molecule H89 Facilitates Mesenchymal Stem Cell‐derived Extracellular Vesicle Release and Optimizes Therapeutic Efficacy in Liver Regeneration
Source: J Extracell Vesicles. 2026 May 9;15(5):e70285. doi: 10.1002/jev2.70285 (PMC13157589; doi:10.1002/jev2.70285)
Supplement: Supplementary file 1 — Supporting Information: jev270285‐Sup‐0001‐SuppMat.docx [file JEV2-15-e70285-s001.docx]

**Supplementary Materials for**

The small molecule H89 facilitates mesenchymal stem cell-derived extracellular vesicles release and optimizes therapeutic efficacy in liver regeneration

Yu Fu^1^* | Yi Ma^1,4^* | Jiajun Zhang^1^* | Liwei Liang^1^ | Ting Li^5^ | Zeyi Guo^6^ | Zhongzhe Li^1^ | Lei Feng^7^ | Yi Wang^8^ | Guolin He^1^ | Shao Li^1^ | Yang Li^1^ | Xiaoping Xu^1#^ | Hui Liao^1#^ | Yi Gao^1,2,3#^

Correspondence to: gaoyi6164@163.com.

This PDF files includes

**Figures S1-S8**

**Tables S1-S3**

**Supplemental Figures:**


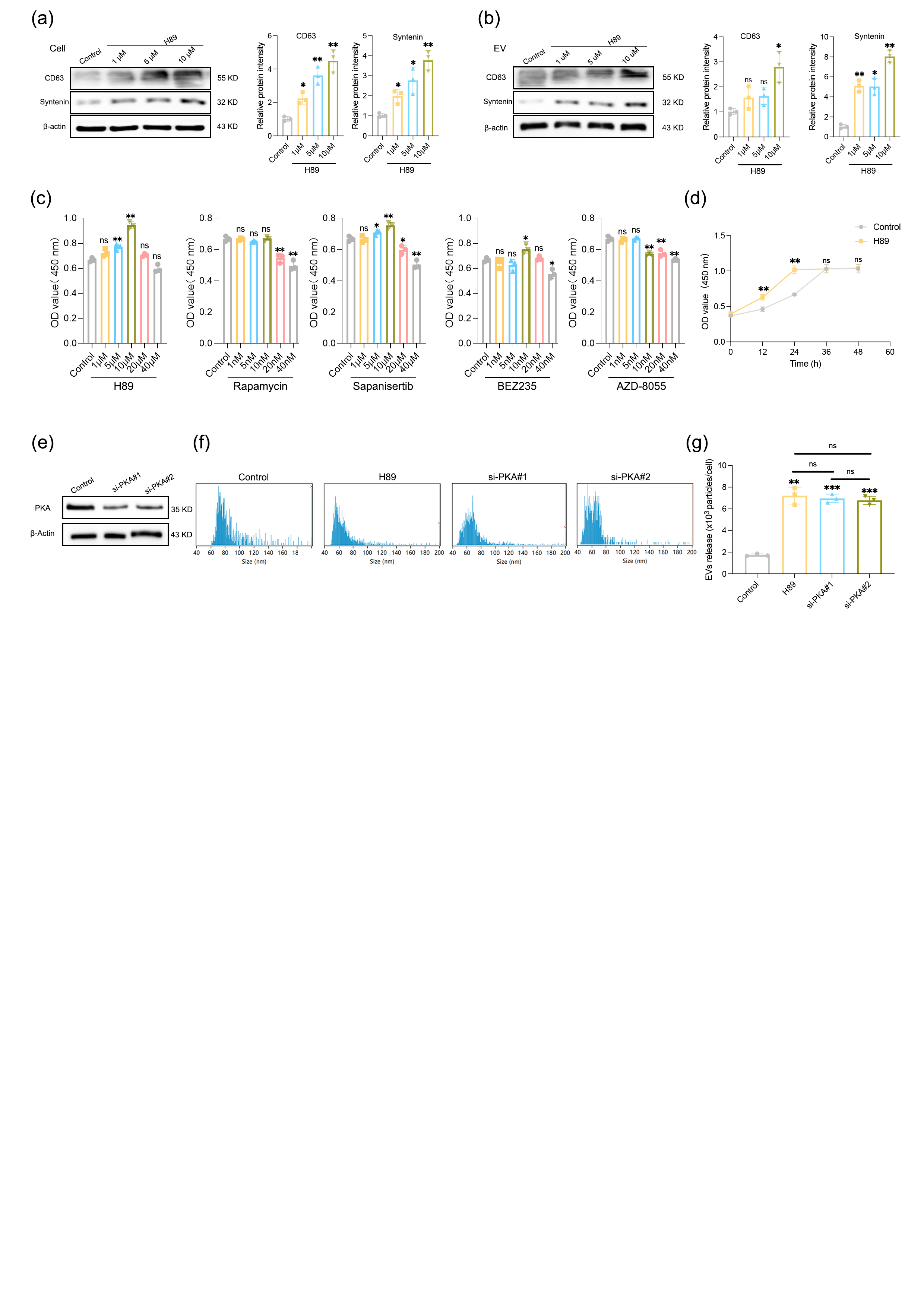


FIGURE S1 Cytotoxicity screening of H89 and evaluation of EVs secretion efficacy. (a) Western blot analysis of H89 doses. (b) Western blot analysis of mTORC1 inhibitors. (c) Cell proliferation assessed by CCK-8 assay under different inhibitors. (d) Proliferation kinetics assessed by CCK-8 assay in hUCMSCs treated with H89 or mTORC1 inhibitors for 24 hours. (d) Proliferation kinetics of hUCMSCs treated with 10 μM H89 for 12-48 h or vehicle control (0.1% DMSO) assessed by CCK-8 assay. (e) Western blot analysis of PKA in hUCMSCs after si-PKA. (f, g) NanoFCM quantifies the particle size distribution and concentration in EVs. All the data are presented as the mean ± SD, n = 3. Unpaired two-tailed Student's t tests were used to test for statistical significance. *p < 0.05, **p < 0.01, ***p < 0.001, ns: not significant.


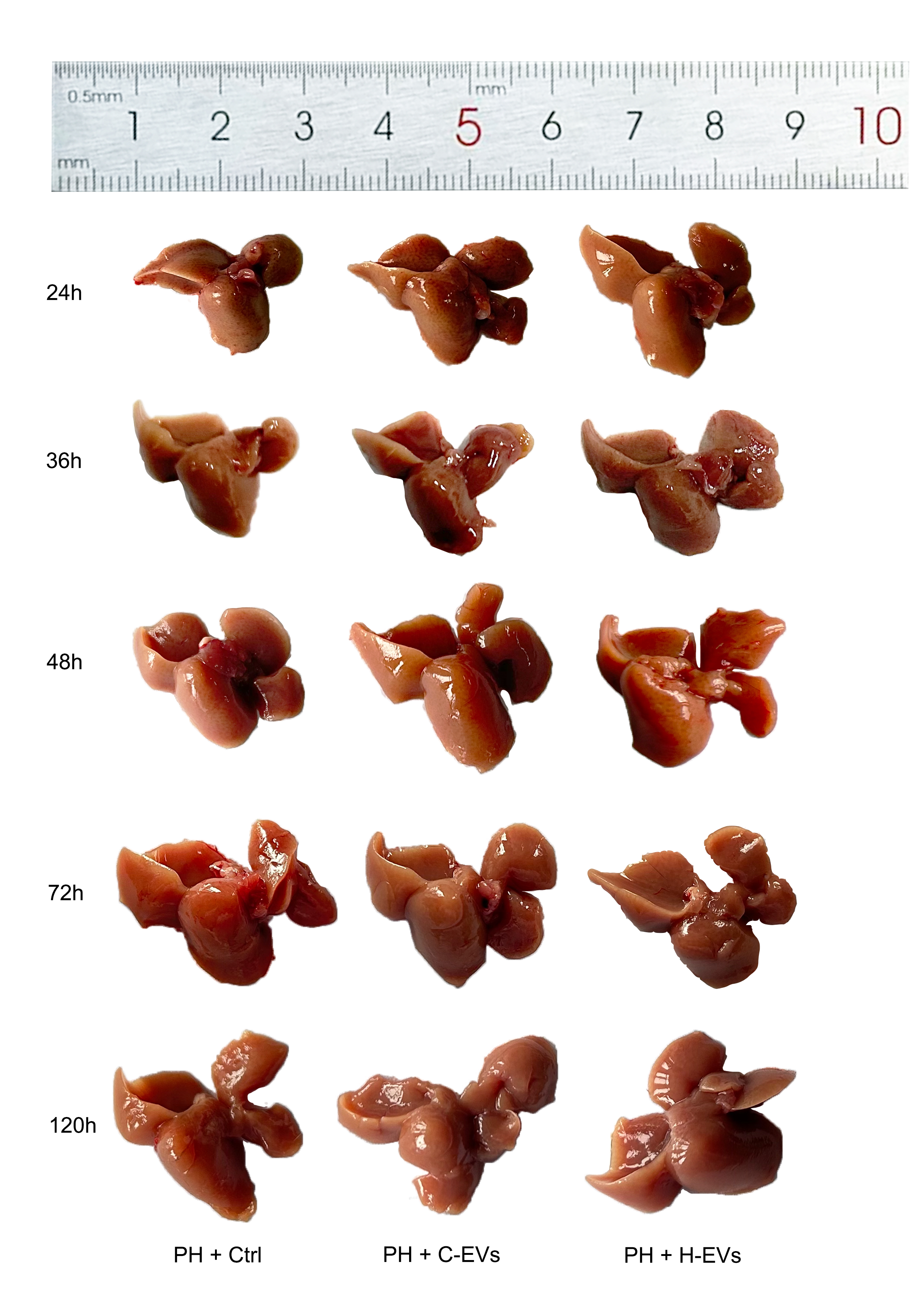
FIGURE S2 EVs derived from H89-pretreated hUCMSCs (H-EVs) promote liver regeneration. Comparison of liver size changes in different treatment groups within 120 hours after hepatectomy (the size of the liver in the image has been standardized for comparison).


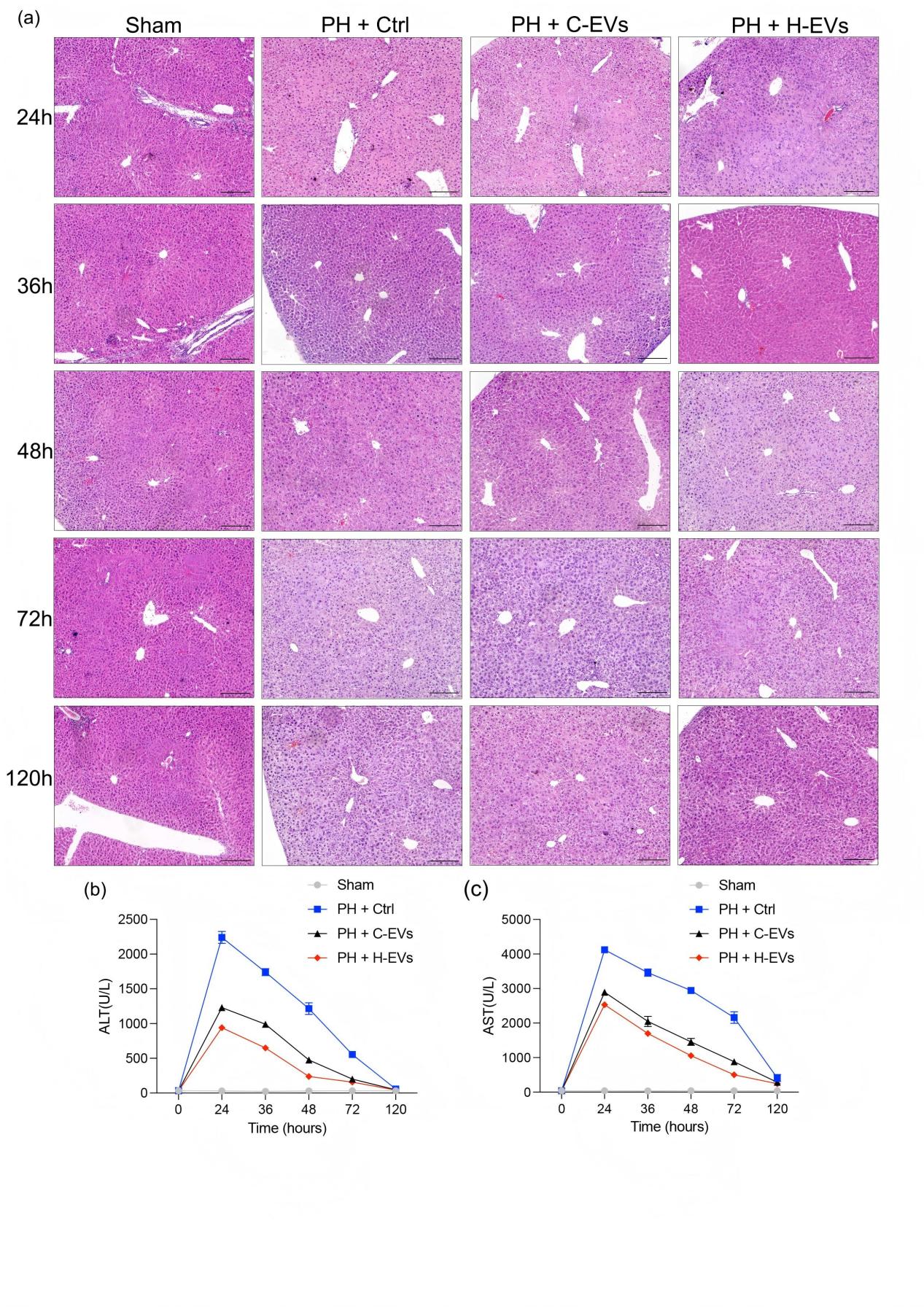


FIGURE S3 EVs derived from H89-pretreated hUCMSCs (H-EVs) alleviate liver injury. (a) H&E staining of liver tissue from partial hepatectomy (PH) mice in the four groups, with a scale bar of 200 μm. (b, c) Serum levels of alanine aminotransferase (ALT) and aspartate aminotransferase (AST). Data were presented as mean ± SD, n = 3.


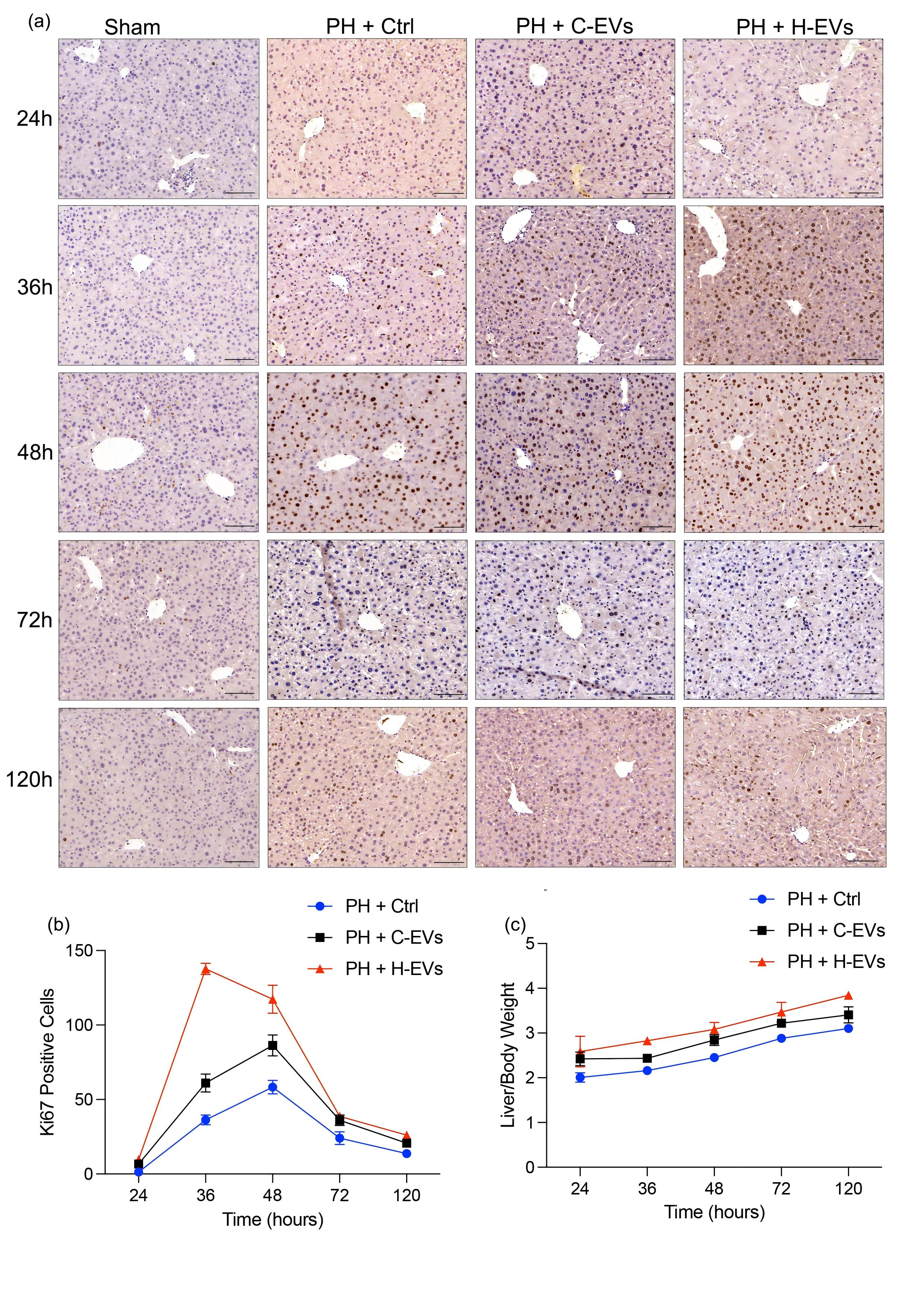
FIGURE S4 EVs derived from H89-pretreated hUCMSCs (H-EVs) promote liver regeneration. (a, b)Ki67 immunohistochemical staining of liver tissue sections from mice at different time points (24h、36h、48h、72h、120h) after partial hepatectomy (PH), (scale bar: 200 μm).（c）Liver weight-to-body weight ratio measurements of mice at different time points after PH surgery. Data were presented as mean ± SD, n = 3.


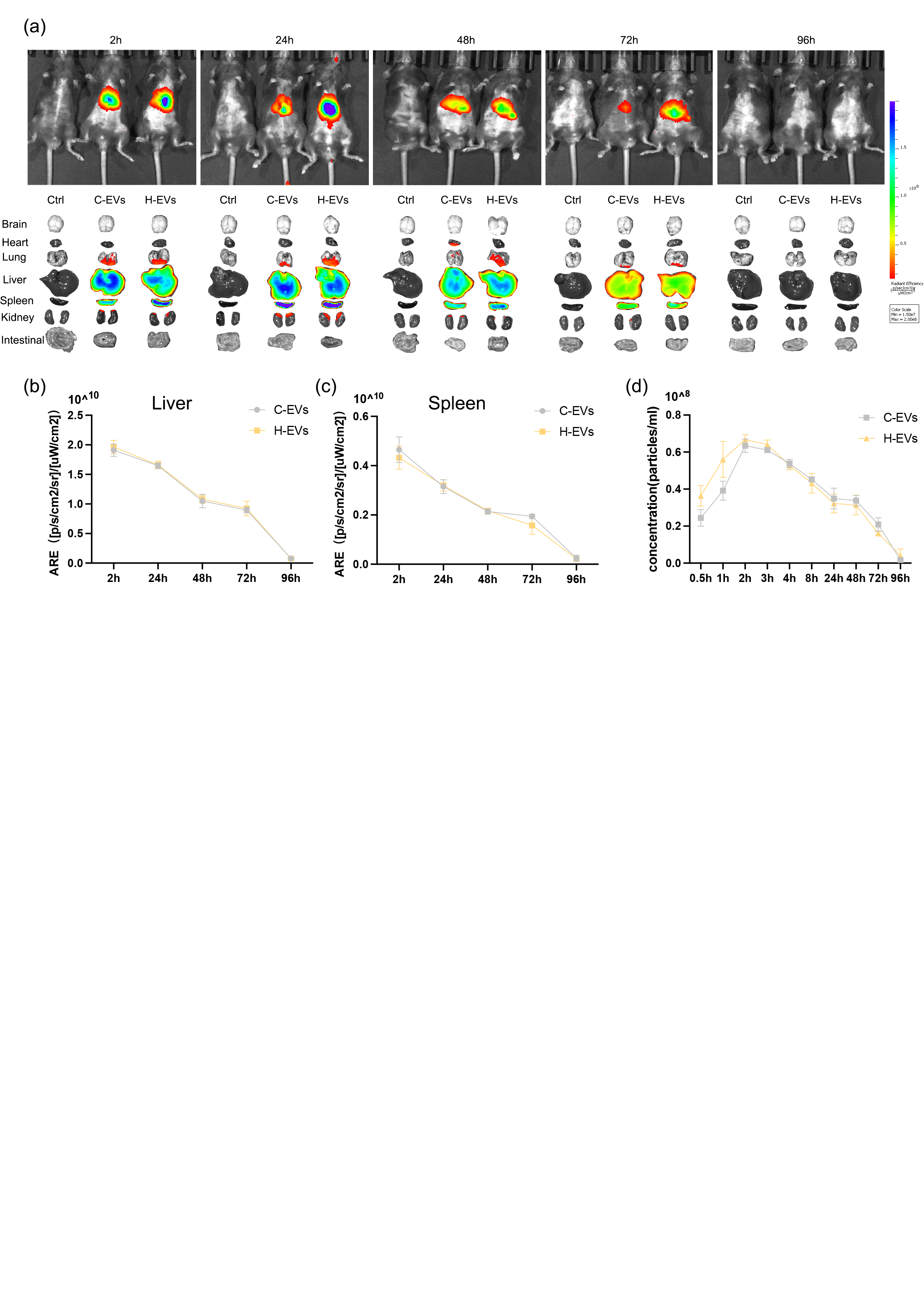


FIGURE S5 The biodistribution of C-EVs and H-EVs. (a) In vivo fluorescence imaging of DiR-labeled EVs (10^9 particles). (b, c) A thorough quantitative analysis of the fluorescence signals in harvested Liver and Spleen. (d) The quantified concentration of CD63-positive particles in the bloodstream over time using a CD63-based immunofluorescence (Nanoview). Data were presented as mean ± SD, n = 3.


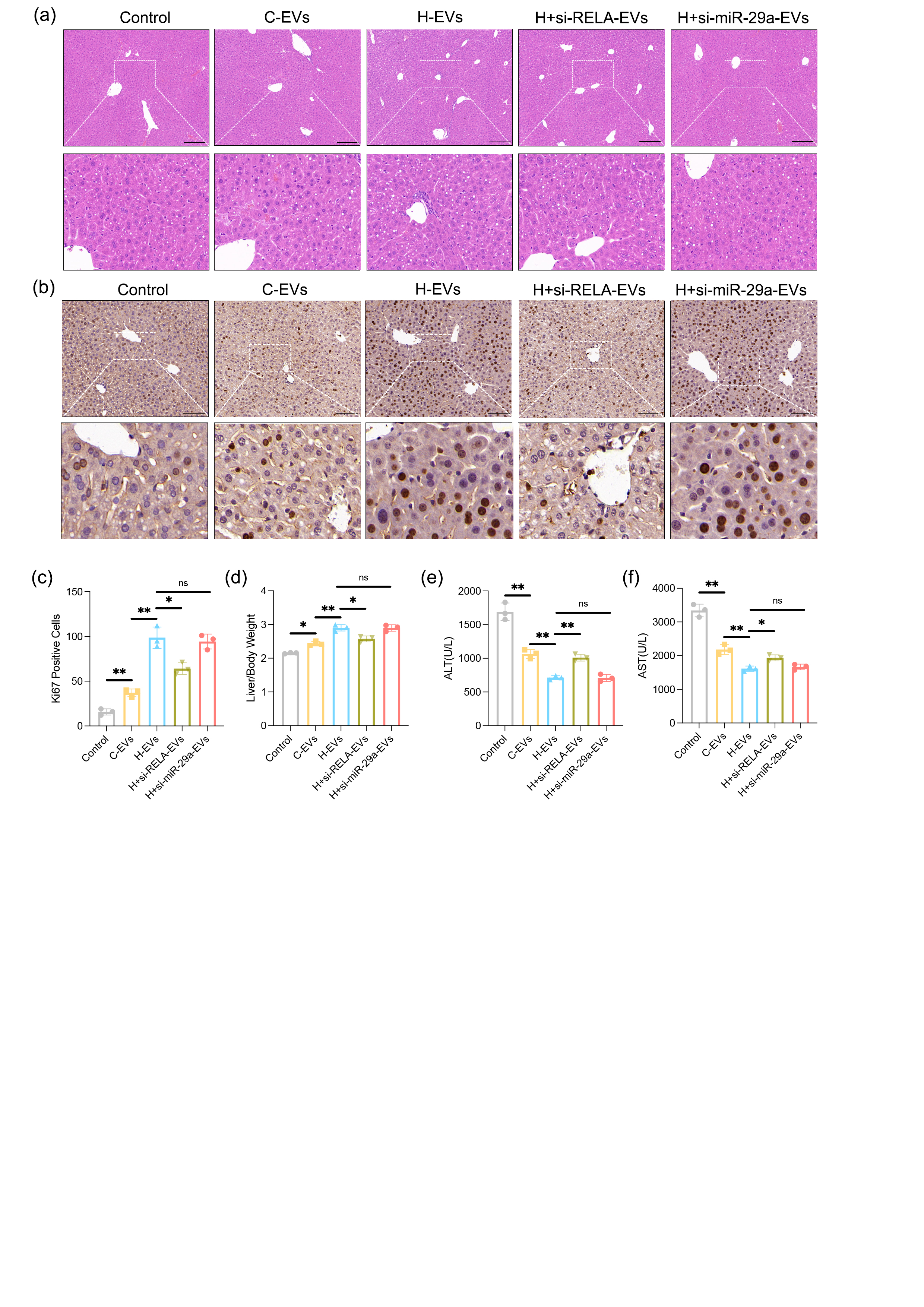


FIGURE S6 EVs derived from H89-pretreated hUCMSCs (H-EVs) promote liver regeneration. (a) H&E staining of liver tissues at 36 hours after PH. Scale bar: 200 μm. (b, c) Ki67 immunohistochemical staining of liver tissue sections from mice at 36 hours after PH. The positive cells were counted in random fields (n = 3). (d) Liver-to-body weight ratio measurements of the mice in the four groups at 36 hours after PH. (e, f) Serum levels of ALT and AST at 36 hours after PH. All the data are presented as the mean ± SD, n = 3. Unpaired two-tailed Student's t test and one-way analysis of variance (ANOVA) were used to test for statistical significance. *p < 0.05, **p < 0.01, ns: not significant.


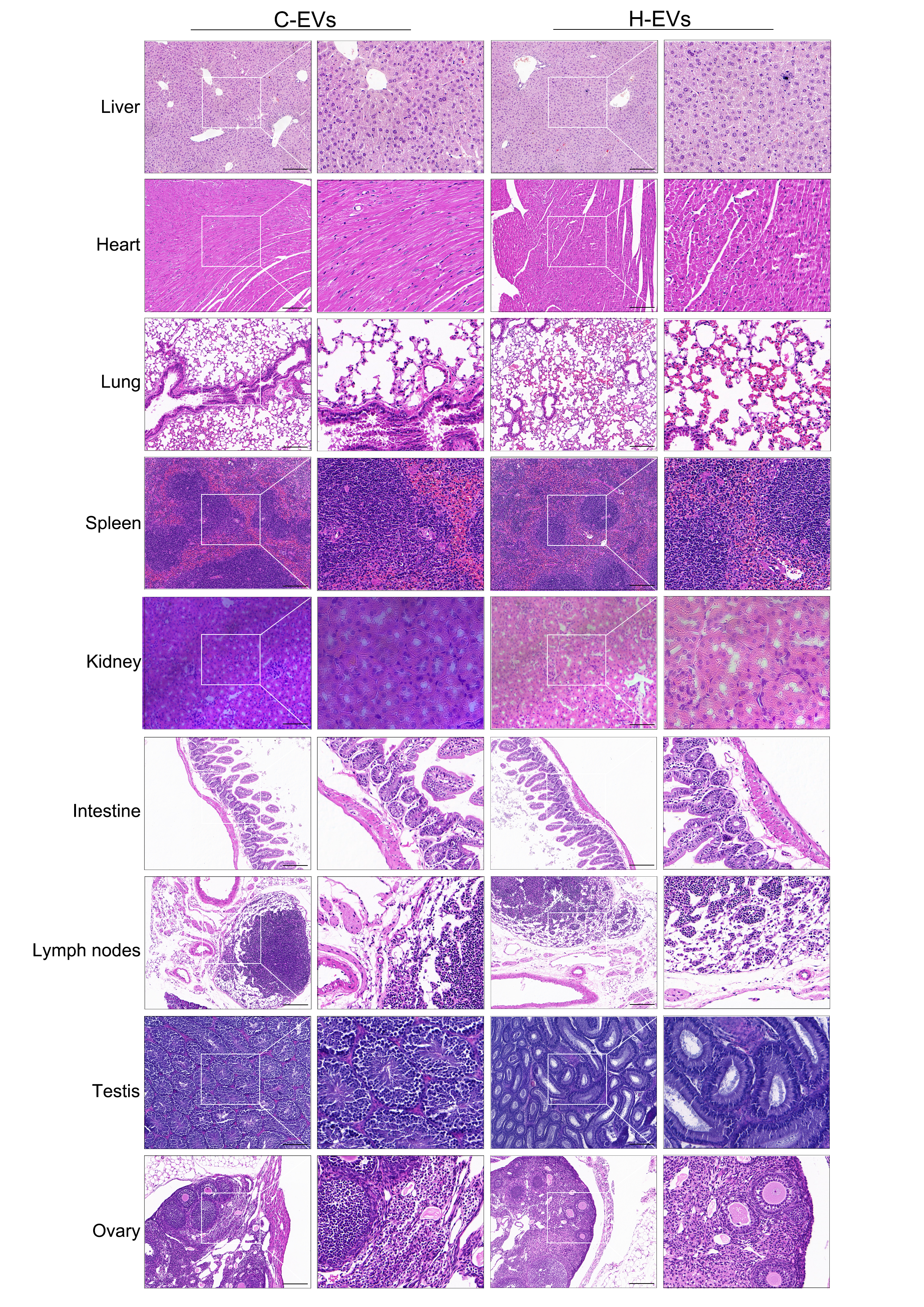
 FIGURE S7 Histological analysis of potential off-target toxicity at 4 weeks following EV administration. H&E staining of major organs, including liver, heart, lung, spleen, kidney, Intestine, Lymph nodes, Testis, Ovary, revealed no significant histopathological abnormalities following systemic administration of H-EVs. Tissue sections showed no evidence of inflammatory infiltration, necrosis, or fibrosis compared to the C-EVs group. These findings suggest that H-EVs did not elicit detectable off-target toxicity under the experimental conditions.


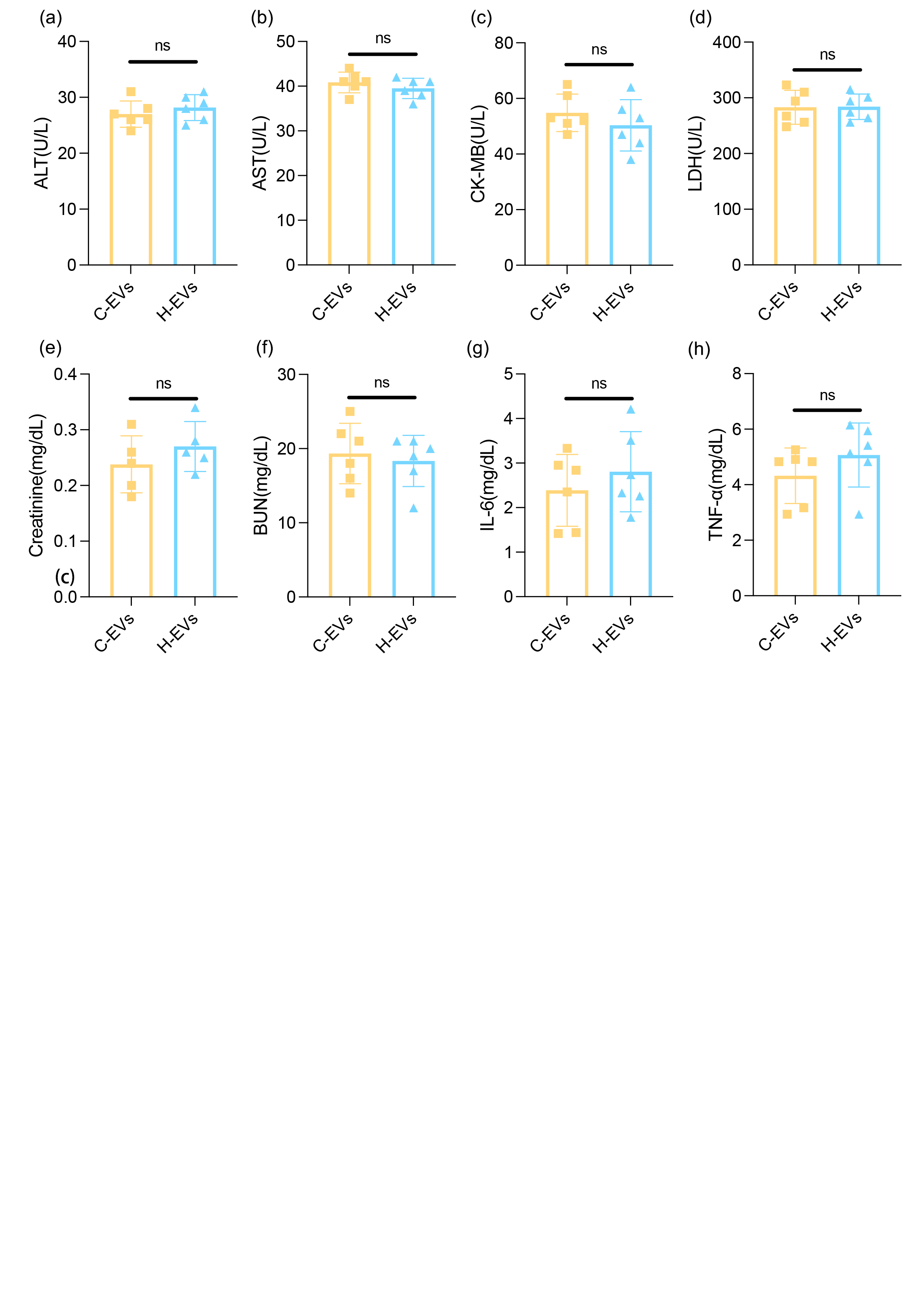


FIGURE S8 Serum biochemical and inflammatory marker analysis at 4 weeks post-EV administration. To assess potential systemic toxicity and inflammatory responses, serum levels of organ damage markers—including alanine aminotransferase (ALT), aspartate aminotransferase (AST), creatine kinase-MB (CK-MB), lactate dehydrogenase (LDH), blood urea nitrogen (BUN), and creatinine—were measured 4 weeks after EV injection. In addition, pro-inflammatory cytokines interleukin-6 (IL-6) and tumor necrosis factor-alpha (TNF-α) were assessed. No significant differences were observed between the H-EVs and C-EVs groups, indicating no apparent hepatic, cardiac, renal, or inflammatory toxicity under the current dosing regimen. All the data are presented as the mean ± SD (n = 6). Unpaired two-tailed Student's t test and one-way analysis of variance (ANOVA) were used to test for statistical significance. ns: not significant.

**Supplemental Tables：**

**Table S1: Information of antibodies**

|  | Source | Catalog number | Dilution |
| --- | --- | --- | --- |
| **Antibodies** |  |  |  |
| CD63 | Abcam | ab59479 | 1:2000 for WB  1:100 for IF |
| cAMP | Abcam | ab76238 | 1:20000 |
| CD9 | Abcam | ab236630 | 1:2000 |
| CD81 | Abcam | ab79559 | 1:500 |
| LC3B | Cell Signaling Technology | #83506 | 1:1000 for WB  1:200 for IF |
| P62 | Cell Signaling Technology | #88588 | 1:1000 |
| mTOR | Cell Signaling Technology | #2983T | 1:1000 |
| p-mTOR | Cell Signaling Technology | #2974T | 1:1000 |
| S6K1 | Cell Signaling Technology | #34475T | 1:1000 |
| RELA | Cell Signaling Technology | #4764T | 1:1000 |
| a-SMA | Cell Signaling Technology | #19245T | 1:1000 |
| TSG101 | Proteintech Group | 28283-1-AP | 1:4000 |
| Calnexin | Proteintech Group | 10427-2-AP | 1:2000 |
| β-actin | Proteintech Group | 60008-1-Ig | 1:5000 |
| SNAP23 | Proteintech Group | 10825-1-AP | 1:1000 |
| SNAP25 | Proteintech Group | 14903-1-AP | 1:60000 |
| SNAP29 | Proteintech Group | 12704-1-AP | 1:20000 |
| VAMP3 | Proteintech Group | 10702-1-AP | 1:2000 |
| VAMP7 | Proteintech Group | 22268-1-AP | 1:4000 |
| GABARAPL1 | Proteintech Group | 11010-1-AP | 1:1000 |

**Table S2. Primers of targeted genes**

| mRNA | Primer pair sequences |
| --- | --- |
| GABARAPL1 | Forward: 5'-ATGAAGTTCCAGTACAAGGAGGA-3'  Reverse: 5'-GCTTTTGGAGCCTTCTCTACAAT-3' |
| VAMP3 | Forward: 5'-GTGGTGGACATAATGCGAGTT-3'  Reverse: 5'-CGTCTGCACGGTCGTCTAAC-3' |
| ATG4B | Forward: 5'-ATGGACGCAGCTACTCTGAC-3'  Reverse: 5'-TTTTCTACCCAGTATCCAAACGG-3' |
| GABARAP | Forward: 5'-AGAAGAGCATCCGTTCGAGAA-3'  Reverse: 5'-CCAGGTCTCCTATCCGAGCTT-3' |
| GABARAPL2 | Forward: 5'-ACTCGCTGGAACACAGATGC-3'  Reverse: 5'-TCTGAGAGCCTGAGACCTTTT-3' |
| ATG2B | Forward: 5'-AACTGCTGACGAATCCTCAGG-3'  Reverse: 5'-GGGGTTCCAGCTAGGTGAGA-3' |
| β-actin | Forward: 5'-AGCGAGCATCCCCCAAAGTT-3'  Reverse: 5'-GGGCACGAAGGCTCATCATT-3' |

**Table S3. Sequences of oligonucleotides used in this study**

| Target name | Oligonucleotide Type | Target sequence (5’→3’) |
| --- | --- | --- |
| GABARAPL1 | siRNA | GCUGCAAGUUCUUGUAUAAUG |
| VAMP3 | siRNA | GAUGUAAUGUUGUCACUAAUU |
| ATG4B | siRNA | GCUACUUCAGCGUCCUCAACG |
| GABARAP | siRNA | GCUAGAACAUUCUAACUAAGA |
| GABARAPL2 | siRNA | GAUUGUUGACAUUGACAAACG |
| ATG2B | siRNA | GGAUGUUGAUGGACAGAUAGA |
| ZNF622 | siRNA | CCUUGAUGAGAUACUACAAAC |
| NDUFS8 | siRNA | AGGAGAAGUUGCUCAACAACG |
| SIGMAR1 | siRNA | GUCUGUUUCUAUUAAUAAAGA |
| RELA | siRNA | GCUGCAGUUUGAUGAUGAAGA |
| UBQLN1 | siRNA | GGAUCAUUCAGCUCAGCAAAC |
| MSRB3 | siRNA | GCUCAAGUUUCAUACUCAACA |
| IL-6 | siRNA | GCUAAAUUCUAGCCUGUUAAU |
| SOD2 | siRNA | GGUCAAAUGUUCCUGUAUAGU |
| RWDD1 | siRNA | GGAGAAUAUUCUUCUGAUAGC |
| APEX1 | siRNA | CCUUAAUUAAGAUCCUCAAUU |
| miR-12116 | miRNA inhibitor | UUAGGCUUCCCCCUCCUCCUGC |
| miR-6830-3p | miRNA inhibitor | CCAAGGAAGGAGGCUGGACAUC |
| miR-425-5p | miRNA inhibitor | AAUGACACGAUCACUCCCGUUGA |
| miR-4716-5p | miRNA inhibitor | AAGGGGGAAGGAAACAUGGAGA |
| miR-6737-5p | miRNA inhibitor | UUGGGGUGGUCGGCCCUGGAG |
| miR-765 | miRNA inhibitor | UGGAGGAGAAGGAAGGUGAUG |
| miR-6864-3p | miRNA inhibitor | GUGAGACUUCUCUCCCUUCAG |
| miR-1207-3p | miRNA inhibitor | UCAGCUGGCCCUCAUUUC |
| miR-1249-3p | miRNA inhibitor | ACGCCCUUCCCCCCCUUCUUCA |
| miR-29a | miRNA inhibitor | UAGCACCAUCUGAAAUCGGUUA |
